# Domain Unknown Function DUF1668-Containing Genes in Multiple Lineages Are Responsible for F_1_ Pollen Sterility in Rice

**DOI:** 10.3389/fpls.2020.632420

**Published:** 2021-01-26

**Authors:** Mitsukazu Sakata, Noriko Takano-Kai, Yuta Miyazaki, Hiroyuki Kanamori, Jianzhong Wu, Takashi Matsumoto, Kazuyuki Doi, Hideshi Yasui, Atsushi Yoshimura, Yoshiyuki Yamagata

**Affiliations:** ^1^ Plant Breeding Laboratory, Faculty of Agriculture, Kyushu University, Fukuoka, Japan; ^2^ Faculty of Agriculture and Marine Science, Kochi University, Nankoku, Japan; ^3^ Institute of Crop Science, National Agriculture and Food Research Organization, Tsukuba, Japan; ^4^ Laboratory of Plant Molecular Breeding, Department of Bioscience, Tokyo University of Agriculture, Tokyo, Japan; ^5^ Graduate School of Bioagricultural Sciences, Nagoya University, Nagoya, Japan

**Keywords:** hybrid incompatibility, reproductive isolation, rice, Oryza, pollen sterility, DUF1668, domain unknown function

## Abstract

Postzygotic reproductive isolation maintains species integrity and uniformity and contributes to speciation by restricting the free gene flow between divergent species. In this study we identify causal genes of two Mendelian factors *S22A* and *S22B* on rice chromosome 2 inducing F_1_ pollen sterility in hybrids between *Oryza sativa* japonica-type cultivar Taichung 65 (T65) and a wild relative of rice species *Oryza glumaepatula*. The causal gene of *S22B* in T65 encodes a protein containing DUF1668 and gametophytically expressed in the anthers, designated *S22B_j*. The *O. glumaepatula* allele *S22B-g*, allelic to *S22B_j*, possesses three non-synonymous substitutions and a 2-bp deletion, leading to a frameshifted translation at the S22B C-terminal region. Transcription level of *S22B-j* and/or *S22B_g* did not solely determine the fertility of pollen grains by genotypes at *S22B*. Western blotting of S22B found that one major band with approximately 46 kDa appeared only at the mature stage and was reduced on semi-sterile heterozygotes at *S22B*, implying that the 46 kDa band may associated in hybrid sterility. In addition, causal genes of *S22A* in T65 were found to be *S22A_j1* and *S22A_j3* encoding DUF1668-containing protein. The allele of a wild rice species *Oryza meridionalis* Ng at *S22B*, designated *S22B_m*, is a loss-of-function allele probably due to large deletion of the gene lacking DUF1668 domain and evolved from the different lineage of *O. glumaepatula*. Phylogenetic analysis of DUF1668 suggested that many gene duplications occurred before the divergence of current crops in Poaceae, and loss-of-function mutations of DUF1668-containing genes represent the candidate causal genetic events contributing to hybrid incompatibilities. The duplicated DUF1668-domain gene may provide genetic potential to induce hybrid incompatibility by consequent mutations after divergence.

## Introduction

Hybrid incompatibilities (HIs), with respect to both intra‐ and interspecific hybridizations, are a widespread mechanism of postzygotic reproductive isolation, which restricts free gene flow between divergent species (Coyne and Orr, 2004). In plants, post-zygotic reproductive isolation occurs throughout the various life cycle stages of hybrids, from fertilization to sexual reproduction (inviability), gametogenesis and fertilization of gametes (sterility) in F_1_ hybrids, and in the subsequent generation (hybrid breakdown; Stebbins, 1950). In cross breeding for genetic improvement in crop species, HI frequently hinders unflagging efforts of hybridization between cultivated and wild species to exploit useful genes and quantitative trait loci (QTLs) from wild genetic resources. Therefore, understanding genetic and molecular basis of HI play an important role in broadening a gene pool for crop improvement and in understanding evolutionary pathway of postzygotic reproductive isolation in crop species. Chromosomal or genic changes that occur during species divergence from common ancestral species are considered to be the main causal events in the evolution of HI (Maheshwari and Barbash, 2011). With respect to genic incompatibility, incompatible combinations of genes, each of which is generally an ancestral and variant allele derived from two reproductively isolated species, combine in the sporophytes or gametophytes of hybrids, resulting in maladaptive phenotypes, leading to inviability, sterility, and/or hybrid breakdown. These incompatible zygotes or gametes with reduced fitness are subsequently eliminated in hybrid populations. However, nucleotide variants causing HI are heterozygous only at birth; thus, a simple genetic model assuming a single locus is insufficient to explain the evolution of HI (Coyne and Orr, 2004). Therefore, it is currently central question of evolutionary genetics of HI that how alleles causing HI can evolve and be maintained in a population without falling into fitness valleys.

Bateson-Dobzhansky-Muller (BDM) incompatibilities are a classical genetic model (Dobzhansky, 1937; Muller, 1942), which proposes that reciprocal genetic changes in divergent species at two or multiple loci allow for the maintenance of new alleles causing HI without negative selection in the intermediate step (Coyne and Orr, 2004; Noor and Feder, 2006). The BDM incompatibilities model emerged based on observations of genetic interactions among multiple loci using a forward genetics approach with hybrid populations derived from inter‐ or intraspecific crosses. The HI system is controlled by the interaction of multiple genes located on different chromosomes, and epistatic complementarity between two genetic loci is exhibited in many plant species such as lettuce (Jeuken et al., 2009), *Arabidopsis* (Bomblies et al., 2007), cotton (Deng et al., 2019), monkey flower (Zuellig and Sweigart, 2018), and wheat (Matsuda et al., 2012). Gene cloning studies have also supported the molecular basis of BDM incompatibilities in plants (Rieseberg and Blackman, 2010; Chen et al., 2016). Gene duplication *via* whole-genome duplication or segmental genome duplication is a primordial event for gene diversification by neofunctionalization and subfunctionalization, as well as for species diversification due to nonfunctionalization (Lynch and Conery, 2000). Reciprocal losses of duplicated genes in divergent plant species were shown to cause F_1_ pollen sterility at the *S27*/*S28* (Yamagata et al., 2010), *DPL1*/*DPL2* (Mizuta et al., 2010), and *DGS1*/*DGS2* (Nguyen et al., 2017) loci, and could lead to hybrid breakdown (Bikard et al., 2009).

When incompatible genes from two divergent populations are closely linked and cause BDM incompatibilities in the hybrid, inheritance of HI appears as a genetic interaction of the gene complex (or haplotype) at a single Mendelian locus in heterozygotes in plants. For example, in rice, intra-subspecific hybrids of cultivated rice carry two adjacent genes encoding F-Box protein and SUMO E3 ligase-like protein at the *Sa* locus to induce pollen sterility (Long et al., 2008), and endoplasmic reticulum stress due to incompatible interactions among heat shock protein Hsp70, an unknown protein with a transmembrane region, and eukaryotic aspartic proteases resulted in embryo sac sterility governed by *S5* (Yang et al., 2012). Recently, the *S1* locus identified in hybrids between *Oryza sativa* and *Oryza glaberrima* that induces F_1_ pollen and embryo sac sterility was found to have originated from a gene complex consisting of *S1A6* and *S1A4* derived from an *O. sativa* allele (Xie et al., 2019) and *S1TPR/SSP* encoding a peptidase-like protein derived from an *O. glaberrima* allele at *S1* (Xie et al., 2017; Koide et al., 2018). As another mechanism, multiple gene copies derived from tandem duplications can acquire new promoter sequences and suppress the expression of genes essential for pollen formation of alternative alleles (Shen et al., 2017).

It has been controversial whether specific protein families or domains are likely to induce HI, although dozens of HI genes have been isolated in plants to date (Rieseberg and Blackman, 2010). HI, including necrosis or weakness, is frequently caused by deleterious interactions of pathogen and insect resistance (*R*) genes in plants (Bomblies and Weigel, 2007), affecting autoimmune responses to ultimately reduce growth, deregulate cell death, and cause sterility (Bomblies et al., 2007; Alcázar et al., 2009; Jeuken et al., 2009; Yamamoto et al., 2010; Chen et al., 2014; Atanasov et al., 2018). In rice, the protease genes involved in hybrid pollen sterility are located at *S1* (Xie et al., 2017; Koide et al., 2018) and involved in embryo sac sterility are located at *S5* (Yang et al., 2012). Genes encoding domain unknown function DUF1618-containing protein at the *Sc* allele on chromosome 3 (Shen et al., 2017) and at *HSA1A* on chromosome 12 in an inter-subspecific hybrid of *O. sativa* (Kubo et al., 2016) were recognized as causal genes of male hybrid sterility. If a specific pattern of functional change or loss of function in a particular domain such as DUF1618 has the potential to induce HI, sporadic independent origins of HI might have occurred across species or genera, which would allow for prediction of the evolution of HI and speciation in geographically isolated species. However, these domains responsible for speciation have been little reported to date.

In the genus *Oryza*, six wild species with an AA genome show an allopatric or sympatric distribution in several continents. *Oryza rufipogon* Griff. and *Oryza nivara* Sharma et Shastry are wild species in South and Southeast Asia, *Oryza longistaminata* A. Chev. & Roehr. and *Oryza barthii* A. Chev. are wild species in Africa, *Oryza glumaepatula* Steud. is a wild species in South America, and *Oryza meridionalis* Ng is a wild species in Australia (Vaughan et al., 2005). Two cultivated species, *O. sativa* L. and *O. glaberrima* Steud., are considered to have been domesticated from *O. rufipogon* and *O. barthii*, respectively. More than 50 loci/QLTs for hybrid sterility have been reported in inter‐ and intraspecific hybrids of rice. Incompatible genotypes of the sporophyte or gametophyte determine sterility, and causal genes at 11 loci have been characterized based on molecular evidence (Chen et al., 2008; Long et al., 2008; Mizuta et al., 2010; Yamagata et al., 2010; Yang et al., 2012; Kubo et al., 2016; Yu et al., 2016; Nguyen et al., 2017; Shen et al., 2017; Koide et al., 2018; Xie et al., 2019).

In backcrossed hybrid progenies derived from a cross between *O. sativa* japonica-type cultivar Taichung 65 (T65) and *O. glumaepatula* accession IRGC105668 in the genetic background of T65, the F_1_ pollen sterility gene *S22* was identified as a Mendelian genetic factor on the short-arm end of chromosome 2 (Sobrizal et al., 2000). The genomic regions of *S22* responsible for pollen sterility were dissected into the two independent genetic loci: *S22A* and *S22B* (Sakata et al., 2014). In the T65 genetic background, plants with the *S22A-T65^+^*/*S22A-glum^s^*|*S22B-T65^+^*/*S22B-T65^+^* genotype (*S22A*_SS plants) showed approximately 50% pollen fertility (semi-sterility) due to sterility of pollen grains carrying the “sterile allele” *S22A-glum^s^*. Similarly, plants carrying the *S22A-T65^+^*/*S22A-T65^+^*|*S22B-T65^+^*/*S22B-glum^s^* genotype (*S22B*_SS plants) showed pollen semi-sterility because of sterility of pollen grains carrying the “sterile allele” *S22B-glum^s^*. The coupling phase linkage of *S22A-glum^s^* and *S22B-glum^s^* on *O. glumaepatula*-derived chromosomal segments could explain the initial identification of *S22* as a single Mendelian factor (Sakata et al., 2014).

In this study, to elucidate molecular players determining postzygotic reproductive isolation between *O. sativa* and *O. glumaepatula*, causal genes of *S22A* and *S22B* for F_1_ pollen sterility between these divergent species were identified by map-based cloning approach. The allelism of another allele in *O. meridionalis* possibly at *S22B* also investigated. Since causal genes of HI both at *S22A* and *S22B* were found to encode DUF1668-containing proteins, which were found to be diversified in Poaceae, phylogenetic analysis of DUF1668 domain in Poaceae was conducted to know evolutionary timing of their occurrence of duplicated copies during divergence of Poaceae.

## Materials and Methods

### Plant Materials and Phenotyping

The backcrossed progenies carrying chromosomal segments derived from *O. glumaepatula* accession IRGC105668 at *S22A* or *S22B* in the *O. sativa* L. cultivar T65 genetic background were developed in a previous study (Sakata et al., 2014). In this study, plants with the genotypes *S22A-T65^+^*/*S22A-glum^s^*|*S22B-T65^+^*/*S22B-T65^+^*, *S22A-T65^+^*/*S22A-T65^+^*|*S22B-T65^+^*/*S22B-glum^s^*, or *S22A-T65^+^*/*S22A-glum^s^*|*S22B-T65^+^*/*S22B-glum^s^* were designated *S22A*_SS, *S22B*_SS, or *S22A+B*_SS plants, respectively. These lines were maintained by marker-assisted selection using the simple sequence repeat (SSR) markers *RM12317* and *RM7451* for *S22A*_SS plants, *RM7033* and *RM279* for *S22B*_SS plants, and *RM12317* and *RM279* for *S22A+B*_SS plants. Phenotypes of *S22A*_SS, *S22A+B*_SS, and *S22B*_SS plants were discriminated according to the morphology of the sterile pollen grains stained with a 1% iodine-potassium iodide solution. Semi-sterile plants, in which almost all of the sterile pollen grains show no staining, stain slightly, and stain dark brown in color and are smaller than normal grains, were classified as *S22A+B*_SS, *S22A*_SS, and *S22B*_SS (Supplementary Figure S1). Transmission frequency of allele was estimated by maximum likelihood method (Supplementary Methods). The homozygous plants for *S22B-glum^s^* and *S22A-T65^+^* obtained in the previous study (Sakata et al., 2014) were used in this study.

### Map-Based Cloning

Total genomic DNA was extracted according to the method described by Dellaporta et al. (1983), with minor modifications. Primer sequences of the polymerase chain reaction (PCR)-based DNA markers used in this study are listed in Supplementary Table S1. Each 15-μl reaction mixture consisted of 50 mM KCl, 10 mM Tris (pH 9.0), 1.5 mM MgCl_2_, 200 mM dNTPs, 0.2 mM primers, 0.75 units of *Taq* polymerase (Takara, Otsu, Japan), and 10 ng genomic DNA template. PCR was performed in a GeneAmp PCR System 9,700 (Applied Biosystems, Foster City, CA, United States). The cycling profile was an initial denaturation step at 95°C for 5 min; 35 cycles of 95°C for 30 s, 55°C for 30 s, and 72°C for 40 s; and a final elongation step at 72°C for 7 min. Amplified products were electrophoresed on a 4% agarose gel in 0.5× TBE buffer. *Agrobacterium*-mediated transformation was conducted as described by Yamagata et al. (2010). In brief, genomic fragments digested by restriction enzymes were cloned into the Ti-plasmid binary vector pPZP2H-lac (Fuse et al., 2001), and were then transformed into *S22A*_SS and *S22B*_SS plants. The copy numbers of transgenes were analyzed by quantitative PCR (qPCR) in an MX3000P QPCR system (Agilent Technologies, Santa Clara, CA, United States) using QuantiTect SYBR Green PCR Kits (Qiagen, Venlo, The Netherlands) according to our previous analysis (Nguyen et al., 2017). The gene models in the mapping region were obtained from MSU Rice Genome Annotation Project Database Release 7 (MSU7; http://rice.plantbiology.msu.edu/) in *O. sativa* cv. Nipponbare Os-Nipponbare-Reference-IRGSP-1.0 and *O. glumaepatula* W1183 accession ALNU02000000 (Jacquemin et al., 2013). The homologous sequences were searched in bl2seq program with cut off score at 1e-50 (Altschul et al., 1990).

### Expression Analysis

For temporal expression analysis of *S22B*, the developmental stages of male gametophytes in T65, *S22B-glum^s^* heterozygotes, and *S22B-glum^s^* homozygotes were observed under a light microscope without staining. Fifty anthers were sampled from the unicellular, bicellular, and mature stages, respectively, which were placed into 1.5-ml tubes and then frozen in liquid nitrogen. Total RNA was extracted using Trizol reagent (Life Technologies Japan, Tokyo, Japan) from the ground anther using a Multibeads shocker (Yasui Kikai, Osaka, Japan). Protein fractions of the Trizol extract were reserved for subsequent western blotting analysis. Extracted RNA was treated with DNase I (Takara, Otsu, Japan) to degrade contaminated genomic DNA. The first-strand cDNA was synthesized from approximately 100 ng of the extracted RNA and then reverse-transcribed using Revertra Ace (Toyobo, Otsu, Japan). Reverse transcription-quantitative PCR (RT-qPCR) was conducted in a MX3000P QPCR system (Agilent Technologies) using QuantiTect SYBR Green PCR Kits (Qiagen). For RT-qPCR of *S22B*, a pair of primers, 5'-CTC TGC CAA CTT CTG CAT CGC CAG G-3'/5'-GCT GAT AAG CTT GTA CAT CTC CGA C-3' and 5'-TGG AGG ATC CAT CTT GGC ATC AT-3'/5'-ACA GCT CCT CTT GGC TTA GCA-3' were used for amplification of *S22B* and the *actin 1* gene (*Os03g0718100*) as an internal control, respectively.

For the promoter-*β*-glucuronidase (*GUS*) assay, genomic sequences of the 1,572-bp region upstream of the initiation codon (ATG) of *S22B-T65^+^* were cloned into the Gateway-entry vector pENTR/TOPO using pENTR Directional TOPO Cloning Kits (Life Technologies Japan). The cloned insert was transferred into the destination vector pGWB3 (Nakagawa et al., 2007) using LR clonase (Life Technologies Japan) to fuse the promoter and *GUS* gene derived from pGWB3. The construct was transformed to T65 by *Agrobacterium*-mediated transformation.

### Subcellular Localization

The coding sequence at *S22B* was amplified from the Nipponbare full-length cDNA clone J023058D10 (AK070727) provided by the National Agriculture and Food Research Organization (NARO), Japan, and was cloned into the Gateway-entry vector pENTR/TOPO (Life Technologies Japan). The *GFP*-fused gene at N-terminal under a control of 35S CaMV promoter was constructed using LR clonase (Life Technologies Japan) into the destination binary vector pGWB6 (Nakagawa et al., 2007). The construct was transformed into T65 by *Agrobacterium*-mediated transformation. The root of the obtained T_0_ plants was observed in a fluorescence microscope (Biozero, BZ-8000, Keyence, Osaka, Japan) after 500 nM of mitochondrial fluorescent dye MitoTracker Red CMXRos staining.

### Western Blotting

For western blotting of the *S22B* product, we prepared a rabbit polyclonal antibody against two synthesized 14-amino acid peptide sequences: N-KLATPLDAGAHDG-C and N-ISGGRKPEQHSLLP-C (Eurofins Genomics, Tokyo, Japan). The antibody specificity was confirmed by western blotting of a 6×His + S22B-T65 fused recombinant protein expressed in *Escherichia coli* strain BL21. The protein fractions extracted by Trizol were mixed with 2× Laemmli sample buffer and 2-mercaptoethanol, and then run on a 12% TGX gel (Biorad, Hercules, CA, United States). The proteins were transferred onto a polyvinylidene difluoride membrane with a 0.2-μm pore size (ATTO, Tokyo, Japan). Goat anti-rabbit IgG (H + L) antibody and horseradish peroxidase-conjugated antibody (Bio-Rad) were used for the secondary antibody reactions, and detection was performed using chemiluminescence detection with Western BLoT Hyper HRP Substrate (Takara Bio, Shiga, Japan).

### Phylogenetic Analysis of DUF1668-Containing Sequences

All protein sequence data deduced in *Setaria viridis*, *Setaria italica*, *Panicum virgatum*, *Botryococcus distachyon*, *Sorghum bicolor*, *Zea mays*, *Botryococcus stacei*, and *Oryza sativa* were downloaded from the Phytozome 12 database (Goodstein et al., 2012). The protein sequences harboring the DUF1668 domain were detected by hidden Markov model searches using hmmsearch software^1^ with a cutoff score of 1e−8. After the amino acid sequences of the DUF1668 domains were aligned using MUSCLE software with default parameters (Edgar, 2004), a phylogenetic tree based on maximum-likelihood inference was constructed in RAxML v. 8.2.8 software (Stamatakis, 2014) and drawn in FigTree v. 1.4.2 software.^2^

## Results

### Map-Based Cloning of *S22B*

**Figure 1:**
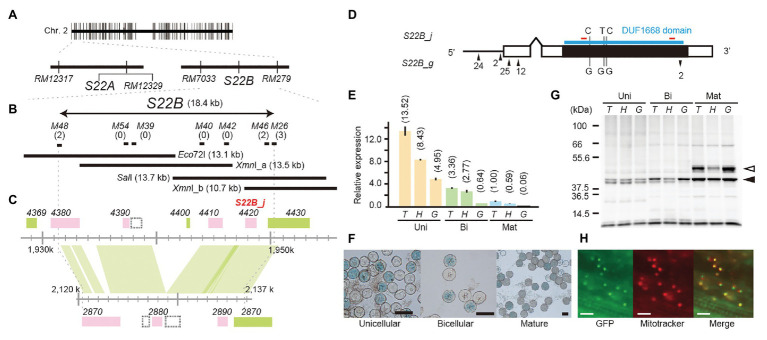
Map-based cloning of *S22B*. **(A)** Two tightly linked genes, *S22A* and *S22B*, independently induce F_1_ pollen sterility. **(B)** High-resolution linkage mapping of *S22B.* Numbers of recombinants between DNA markers and phenotypes are shown in parentheses. The restriction fragments *Eco*72I, *Xmn*I_a, *Sal*I, and *Xmn*I_b were used for transformation experiments. **(C)** Genomic structures on the reference genome assembly of *Oryza sativa* cv. Nipponbare Os-Nipponbare-Reference-IRGSP-1.0 (Kawahara et al., 2013) and *Oryza glumaepatula* W1183 accession ALNU02000000 (Jacquemin et al., 2013). Green and pink rectangles represent annotated genes encoded in the plus and minus chain of the reference sequences, respectively. The hatched rectangles represent predicted transposable elements. **(D)** Nucleotide variations on the 1.5-kb promoter (black horizontal bar) and coding sequence (black rectangles) between *S22B_j* and *S22B_g*. Up and down arrowheads represent nucleotide insertions and deletions, respectively, in *S22B_g* against *S22B_j*. The DUF1668 domain is shown as a blue line. Red bars show the location of peptide sequences to develop S22B antibody. **(E)** Reverse transcription-quantitative PCR (RT-qPCR) of *S22B* in the anthers during male gametogenesis for each genotype at *S22B*. Relative expression compared to the transcript in homozygotes for T65 on mature stage is represented in parentheses. **(F)** Promoter-glucuronidase (*GUS*) assay using the *S22B* promoter. T, H, and G represent genotypes homozygous for the T65 allele, heterozygous, and homozygous for IRGC105668, respectively. Scale bar: 50 μm. **(G)** Western blotting of S22B protein in the anthers. Total protein levels among samples were adjusted according to the numbers of collected anthers from 50 spikelets. White and black arrowheads represent the two major bands mentioned in text with the estimated mass of 37 and 46 kDa, respectively. **(H)** Subcellular localization of N-GFP-S22B in root cells of the stable transformant (T_0_). Scale bar: 20 μm.

To narrow down the candidate region of *S22B*, we conducted high-resolution mapping of *S22B* in the BC_4_F_6_ population in which both *S22A* and *S22B* segregated (*n* = 7,424; Figure 1). Since *S22B* was previously mapped between the SSR markers *RM12329* and *RM279* (Sakata et al., 2014), 308 recombinants obtained between the SSR markers *RM12329* and *RM279* were screened by genotyping at the seedling stage. Linkage analysis of the 308 recombinants demonstrated that five recombinants (9-7, 23-2, 28-1, 38-3, and 39-7) were the most informative plants to map *S22B* within 18.4 kb of the genomic region in the reference sequence Nipponbare between DNA markers *M48* and *M46* (Supplementary Figure S2; Figure 1B). For the complementation test of *S22B*, 13.1-kb *Eco*72I, 13.5-kb *Xmn*I (*Xmn*I_a), 13.7-kb *Sal*I, and 10.7-kb *Xmn*I (*Xmn*I_b) fragments of the Nipponbare genomic fragment derived from BAC clone OSJNBa008C13 were introduced into *S22B*_SS plants by *Agrobacterium*-mediated transformation (Figure 1B). In the non-transgenic progeny of *S22B*_SS, a reduced transmission efficiency (*k*) of *S22-glum^s^* alleles (*k* = 0.11) *via* pollen from the theoretical transmission (*k* = 0.5) was observed, which was attributed to the sterility of pollen grains carrying *S22-glum^s^* (Table 1). Similarly, the T_1_ generation derived from the T_0_ plants transformed by the *Eco*72I, *Xmn*I_a, and *Xmn*I_b fragments showed segregation distortion of genotypes at *RM7033* linking to *S22B* because of reduced transmission of *S22-glum^s^* (Table 1). Meanwhile, transformation of the *Sal*I fragment significantly altered the segregation of the genotype at *RM7033* in the T_1_ generation (Table 1), suggesting that pollen grains harboring *S22-glum^s^* recovered pollen fertility by the transformation of the *Sal*I fragment. Pollen fertility of the T_1_ plants derived from the T_0_ plant with the *Sal*I fragment or *Xmn*I_a fragment was observed (Supplementary Figure S3). The two T_1_ lines, 6 and 7, were derived from the two independent T_0_ plants carrying a single copy and more than two copies of the *Sal*I fragment, respectively. The heterozygotes at *S22B* harboring *Sal*I fragment showed recovered pollen fertility as compared with null segregants of heterozygotes or the heterozygotes harboring *Xmn*I_a fragment (Supplementary Figure S3). In the T_1_ line 6, transgene segregated at one locus and the heterozygous plants at *S22B* carrying two, one, and zero copies of the *Sal*I fragment showed more than 90%, approximately 75, and 50% of pollen fertility, respectively, whereas the transformation of the *Xmn*I_a fragment did not recover pollen fertility in T_1_ as a negative control (Supplementary Figure S4). *LOC_Os02g04420* was predicted to be located on the *Sal*I fragment but not on the other genomic fragments in the MSU7. These data demonstrated that the causal gene of *S22B* is *LOC_Os02g04420*, which encodes a protein containing DUF1668, designated *S22B_j* (Figures 1C,D). The genomic sequences of *S22B* including 2,500 bp of upstream region from the transcription initiation site are identical to those of Nipponbare. Sequencing of the *O. glumaepatula* (Acc. IRGC105668) BAC clone GL47D11 showed that the *O. glumaepatula* allele *S22B-g*, allelic to *S22B_j*, possesses three non-synonymous substitutions CCG(P) > GCG(A), TTC(F) > GTC (V), and CCC(P) > GCC(A) and a 2-bp deletion, leading to a frameshifted translation at the S22B C-terminal region (Supplementary Data 1). Many nucleotide substitutions at the promoter and first exon regions were also observed (Figures 1C,D; Supplementary Data 1). The *S22B* sequences of T65 and IRGC105668 were deposited to DNA databank of Japan (DDBJ; LC596092 and LC596094).

**Table 1:** Frequency of genotypes at *RM7033* in the T_1_ generation for the complementation test of *S22B*.

Construct	Recipient	Frequency of genotypes at *RM7033* at T_1_ ^1^					
		*TT*	*TG*	*GG*	Total	*p*	*k*
*Eco*72I	*S22B*_SS	13	20	0	33	0.32	0
*Xmn*I_a	*S22B*_SS	26	21	5	52	0.52	0.16
*Sal*I	*S22B*_SS	18	41	19	78	0.00017^***^	0.51
*Xmn*I_b	*S22B*_SS	15	12	2	29	0.74	0.12
Non-transgenic	*S22B*_SS	42	43	5	90	-	0.11

1 *TT*, *TG*, and *GG* are homozygous for *S22B-T65^+^*, heterozygous, and homozygous for *S22B-glum^s^*, respectively. Values of *p* for independence of the segregation ratio in the T_1_ generation were tested using Fisher’s exact test at empirical segregation ratios of 42:43:5 for *TT*, *TG*, and *GG* plants derived from self-pollination of non-transgenic *S22B*_SS plants. *k* represents the transmission efficiency of *S22B-glum^s^ via* pollen from maximum-likelihood estimation. ***represents significance at 0.1% significance level.

### Molecular Characterization of *S22B*

Expression of *S22B* from the anthers at the unicellular, bicellular, and mature stages was investigated by RT-qPCR among *S22B-T65^+^* homozygotes, *S22B-glum^s^* heterozygotes, and *S22B-glum^s^* homozygotes. Transcript levels gradually decreased during the progression of post-meiotic male gametogenesis (Figure 1E). At the unicellular, bicellular, and mature stages, the *S22B* expression level was elevated in the order *S22B-T65^+^* homozygotes, heterozygotes, and *S22B-glum^s^* homozygotes, suggesting that transcription of *S22B-glum^s^* was not as active as that of *S22B-T65^+^*. The promoter-*GUS* assay was conducted in T_0_ plants with 1.5 kb of endogenous genomic sequences upstream from the initial codon of *S22B-T65* in the unicellular, bicellular, and mature pollen stages (Figure 1F). Half of the pollen grains displayed GUS signals in T_0_ transgenic plants carrying a single copy of the transgene, but no GUS signals were detected in the anther tissues, demonstrating that *S22B* is gametophytically expressed in the haploid generation. Together, the results from these expression analyses suggested that the level of gametophytic transcripts of *S22B-T65^+^* or *S22B-glum^s^* did not solely determine the fertility of pollen grains carrying *S22B-T65^+^* or *S22B-glum^s^* alleles.

Expression of S22B protein was investigated by western blotting using anti-S22B antibody in the anthers at the unicellular, bicellular, and mature stages (Figure 1G). The deduced molecular mass of S22B-T65^+^ and S22B-glum^s^ proteins was expected to beapproximately 39 kDa based on the coding nucleotide sequences. The density of approximately 37 kDa band estimated in western blotting (37 kDa) gradually increased during the pollen stage. Another major band at a molecular mass probably 46 kDa (46 kDa band) appeared only at the mature stage, and was reduced in the *S22B-glum^s^* heterozygotes as compared with those of homozygotes for *S22B-T65^+^* or *S22B-glum^s^* alleles. Although the levels of *S22B* transcripts were reduced in homozygotes for the *S22B-glum^s^* allele (Figure 1E), protein levels between homozygotes for the *S22B-T65^+^* or *S22B-glum^s^* allele were comparable (Figure 1G), implying that the transcript level was sufficient for expression of S22B protein and could be adjusted *via* feedback regulation.

The intracellular localization of S22B was investigated on root cells of the stable transformant (T_0_) transformed by the construct of 35SCaMV prom::N-GFP-S22B protein (Figure 1H). Green fluorescent protein (GFP) signals were colocalized with the mitochondrial fluorescent dye MitoTracker Red CMXRos on root cells. The program for subcellular localization prediction in plant cells, TargetP-2.0 (Almagro Armenteros et al., 2019), MitoFate (Fukusawa et al., 2015), and Localizer (Sperschneider et al., 2017) did not find apparent prediction of localization to the mitochondria. WolfPSORT programs (Horton et al., 2007) weakly suggested that S22B possesses mitochondrial-targeting peptide sequences.

### Map-Based Cloning of Gametophytic Factors at *S22A*

**Figure 2:**
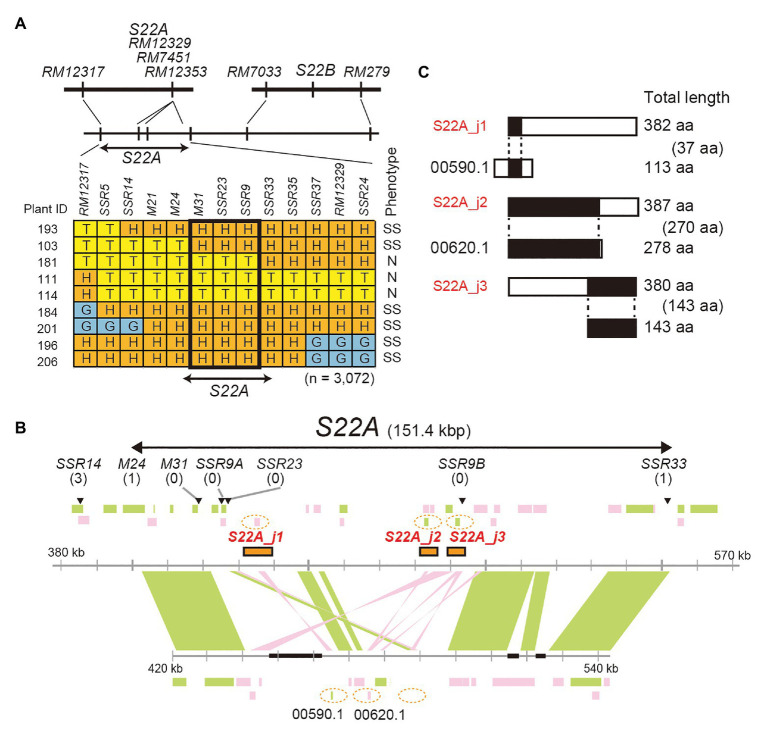
Map-based cloning of *S22A*. **(A)** High-resolution linkage mapping of *S22A*. T, H, and G represent genotypes homozygous for the T65 allele, heterozygous, and homozygous for IRGC105668, respectively. N and SS represent a pollen phenotype of normal fertility and semi-sterility, respectively. **(B)** Genomic structures on the reference genome assembly of *O. sativa* cv. Nipponbare Os-Nipponbare-Reference-IRGSP-1.0 and *O. glumaepatula* W1183 accession ALNU02000000. Three restriction fragments, *Acc*65I_a, *Acc*65I_b, and *Acc*65I_c, including *S22A_j1*, *S22A_j2*, and *S22A_j3*, respectively, were used for transformation experiments. These fragments are shown as orange rectangles. Black boxes represent a sequence gap in the reference sequence. Numbers of recombinants between DNA markers and phenotypes are shown in parentheses. Green and pink rectangles represent annotated genes encoded in the plus and minus chain of the reference sequences, respectively. **(C)** Putative pseudogenes for DUF1668-containing protein in the *O. glumaepatula* genome at *S22A*. A similarity search using the BLASTn program revealed that only parts of DUF1668-containing protein S22A_j1, S22A_j2, and S22A_j3 showed sequence similarity in the *O. glumaepatula* genome. *S22A_j1* and *S22A_j2* appear to be allelic to *OGLUM02G00590.1* and *OGLUM02G00620.1*, respectively. However, DUF1668 domains were lost in the *O. glumaepatula* genome. Alignment lengths between proteins are shown in parentheses.

High-resolution mapping of *S22A* was conducted using the BC_4_F_7_ population (*n* = 3,072) derived from the BC_4_F_6_ plants heterozygous at the *S22A* genomic region between the SSR markers *RM12317* and *RM12350*, and homozygous for T65 at *S22B* in the genetic background of T65 (*S22A*_SS plants; Figure 2). Our previous study revealed that *S22A*_SS plants showed approximately 50% pollen sterility due to the sterility of pollen grains harboring *S22A-glum^s^*; consequently, homozygotes for *S22A-T65^+^*, heterozygotes, and homozygotes for *S22A-glum^s^* segregated at a 1:1:0 ratio (Sakata et al., 2014). The genomic region responsible for *S22A* was delimited within a 151.4-kb region between the DNA markers *M24* and *SSR33* (Figure 2A). Homozygous plants for *S22A-glum^s^* were not obtained in the high-resolution mapping population, suggesting that male gametophytes with *S22A-glum^s^* in heterozygotes are completely sterile. Within the candidate genomic region, three gene models, *LOC_Os02g01790* designated *S22A_j1*, *LOC_Os02g01870* designated *S22A_j2*, and *LOC_Os02g01900* designated *S22A_j3*, were found to harbor DUF1668 in the reference genomic sequence of Nipponbare based on a Pfam search (Figure 2B). We speculated that the *S22A-T65^+^* allele has a function to gametophytically provide fertility to pollen grains carrying the *S22A-T65^+^* allele in spite of the haploid genotype, as in the case of *S22B*. Three *Acc*65I-digested genomic fragments *Acc*65I_a, *Acc*65I_b, and *Acc*65I_c, containing *S22A_j1*, *S22A_j2*, or *S22A_j3*, respectively, were subcloned from the Nipponbare BAC clone OSNBb0096M07 and transformed into *S22A*_SS plants. The T_1_ population for *S22A_j1* showed segregation of homozygous genotypes for *S22A-glum^s^*, demonstrating that pollen grains carrying the *S22A-glum^s^* allele restored pollen fertility due to the effect of the *S22A_j1* transgene (Table 2; Supplementary Table S2). The transgene in the T_1_ population for *S22A_j3* resulted in segregation of two homozygotes for *S22A-glum^s^*, the frequency of which was lower than that in the T_1_ population in *S22A_j1*. Moreover, the homozygous plants for *S22A-glum^s^* were not observed in the T_1_ population derived from the T_0_ plants transformed by *S22A_j2*. If *S22A_j2* has equivalent function to *S22A_j1*, non-segregation of the homozygous plants for *S22A-glum^s^* means *S22A_j2* does not function to restore pollen fertility with *S22A-glum^s^*. Meanwhile, if *S22A_j2* has a partial function to restore the sterility as shown in transformation of *S22A_j3*, the number of T_1_ individuals in this population might not have been sufficient to detect complementation of gene function. Therefore, further study is necessary to elucidate the function of this gene. Eventually, it was concluded that transgenes encoding protein with the DUF1668 domain derived from *S22A-T65^+^*, at least *S22A_j1* and *S22A_j3*, gametophytically restored the fertility of pollen grains carrying the transgene. The amino acid sequences of *S22A_j1*, *S22A_j2*, and *S22A_j3* deduced from *O. glumaepatula* genomic sequences lacked DUF1668, suggesting that these three alleles at *S22A-glum^s^* lost their function as a DUF1668-harboring protein (Figure 2C).

**Table 2:** Complementation test of *S22A* by transmission analysis at *SSR23*.

Transgene	Genotype at *SSR23* ^1^				
	*TT*	*TG*	*GG*	Total	*k*
*S22A_j1*	54	57	17	128	0.239
*S22A_j2*	19	20	0	39	0.000
*S22A_j3*	46	55	2	103	0.042
Empty vector	35	41	0	76	0.000

1 *TT*, *TG*, and *GG* are homozygous for *S22A-T65^+^*, heterozygous, and homozygous for *S22B-glum^s^*, respectively. *k* represents the transmission efficiency of *S22A-glum^s^ via* pollen from maximum-likelihood estimation. Segregation of the homozygous plants for *S22B-glum^s^* (underlined) represent transmission of *S22B-glum^s^* due to recovery of pollen fertility of pollen grains carrying *S22B-glum^s^*.

### Independent Origin of Sterile Alleles in *O. meridionalis* at *S22B*

During the process of development of introgression lines of *O. meridionalis* accession W1625 (MER-ILs) in the genetic background of T65 (Yoshimura et al., 2010), pollen semi-sterile plants carrying W1625 chromosomal segments on the short arm of chromosome 2 with T65 cytoplasm were observed in the BC_4_F_2_ population (Supplementary Figure S5). Linkage mapping using the BC_4_F_3_ progeny demonstrated that semi-sterile and normal fertile pollen completely co-segregated to *RM7033*, and one single Mendelian factor close to *RM7033* located in the genomic region between SSR markers *RM7451* and *RM5984* controls pollen sterility in this population. This genetic factor was designated as *S22-mer*. Further genetic dissection of the Mendelian factor *S22-mer* using 753 plants of the mapping population indicated that *S22-mer* is located between *SSR24* and *RM5984* containing the *S22B* locus. The *O. meridionalis* allele at *LOC_Os02g04420* corresponding to *S22B_j* in *O. sativa* was designated *S22B_m*. Sequencing of the *O. meridionalis* BAC clone OMERIa-82O19 containing *S22B_j* allelic region showed that *O. meridionalis* has lost the upstream gene region, including the promoter, transcription initiation site, and N-terminal region of the DUF1668-containing protein (Supplementary Data 1). However, *S22B_m* does not possess the non-synonymous substitutions and a 2-bp deletion found in *O. glumaepatula* (Supplementary Data 1) except the CCG (P) > GCG (A) mutation. These results suggest that *S22B_m* is a loss-of-function allele and evolved from the different lineage of *O. glumaepatula*. The *S22B* sequence of W1625 was deposited to DDBJ (LC596093).

### Diversity Analysis of DUF1668

**Figure 3:**
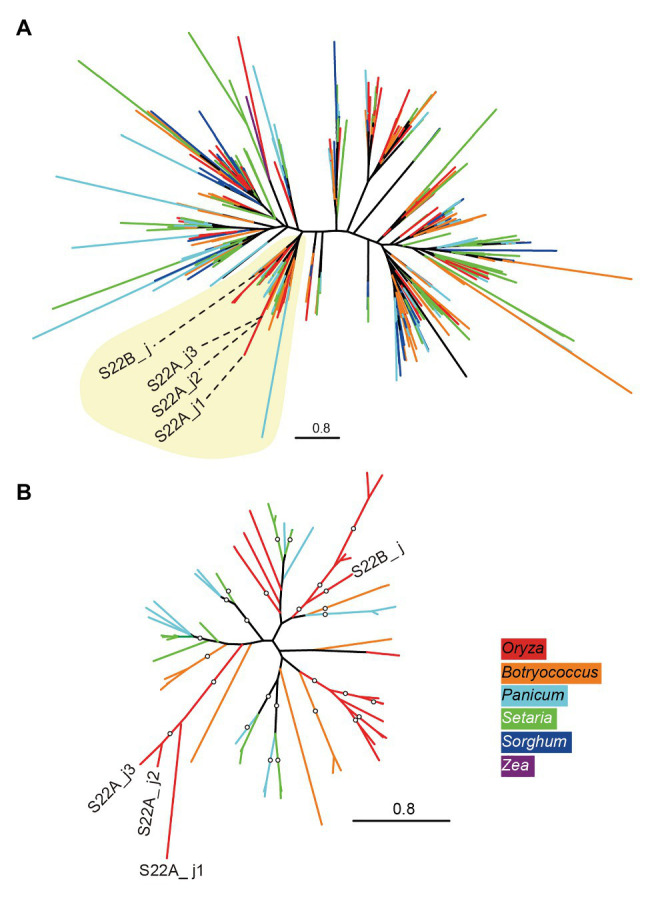
Phylogenetic tree of DUF1668 domain in Poaceae species. **(A)** Maximum-likelihood trees showing the phylogenetic relationships of DUF1668 domain found in Poaceae species were constructed. The colors of external nodes (leaves) indicate the genus of DUF1668 domain members: red (*Oryza*), orange (*Botryococcus*), light blue (*Panicum*), green (*Setaria*), dark blue (*Sorghum*), and purple (*Zea*). Clade 1 (shaded in yellow) includes the DUF1668 members from the genera *Oryza*, *Botryococcus*, *Panicum*, and *Setaria*. **(B)** Reconstructed tree of DUF1668 members belonging to Clade 1. The branches supported by more than 75% bootstrap values are indicated by circles.

A BLASTP search using the amino acid sequences of *S22B_j* as a query in the proteome of angiosperm species in the Phytozome 12 database (Goodstein et al., 2012) found homologous sequences of proteins only in Poaceae species, including the PACMAD clade species *S. viridis*, *S. italica*, *P. virgatum*, *S. bicolor*, and *Z. mays*, and the BOP clade species *B. distachyon*, *B. stacei*, and *O. sativa* (Supplementary Figure S6). Phylogenetic relationships based on DUF1668-containing sequences in Poaceae species revealed that each genome showed more than 40 copies of DUF1668-containing genes except for *Z. mays* (Supplementary Figure S6). The phylogenetic tree of DUF1668 domain sequences did not exhibit apparent monophyletic branches by species (Figure 3A), suggesting that active gene duplication and diversification of multiple copies of DUF1668-containing genes likely started before the diversification of Poaceae. However, *S22B_j*, *S22A_j1*, *S22A_j2*, and *S22A_j3* were included in a single clade (Clade 1) harboring other members from *Setaria*, *Panicum*, *Botryococcus*, and *Oryza* species (Figure 3, yellow-shaded area). Reconstruction of the phylogenetic tree of DUF1668 members in Clade 1 revealed that DUF1668 sequences deduced from *S22A_j1*, *S22A_j2*, and *S22A_j3* formed a monophyletic clade, but *S22B_j* was found to have an independent origin from the clade containing *S22A_j1*, *S22A_j2*, and *S22A_j3* (Figure 3B).

## Discussion

Here, we revealed that the genes *S22B_j*, *S22A_j1*, and *S22A_j3* encoding DUF1668-containing protein are causal genes of F_1_ pollen sterility controlled by *S22A* or *S22B* in hybrids derived from a cross between *O. sativa* cultivar T65 and *O. glumaepatula* accession IRGC105668. Our previous genetic analysis demonstrated that *S22A* and *S22B* independently induced pollen semi-sterility on heterozygotes, and that pollen grains carrying the sterility allele *S22A-glum^s^* and *S22B-glum^s^* in *S22A*_SS and *S22B*_SS plants are, respectively, sterile (Sakata et al., 2014). The transformation of *S22B_j* to *S22B*_SS plants recovered the fertility of pollen grains harboring sterile alleles (Supplementary Table S3) and transmission of the sterile allele *via* pollen increased (Table 1). Although the sterile allele *S22A-glum^s^* had never transmitted *via* pollen in the high-resolution linkage mapping, the transformation of *S22A_j1* or *S22A_j3* to *S22A*_SS plants also archived transmission of the sterile allele *via* pollen (Table 2), demonstrating recovery of the fertility of pollen grains harboring sterile alleles by the transgene. Since *S22B_j*, *S22A_j1*, and *S22A_j3* are linked in the coupling phase, *S22A* and *S22B* were previously considered to represent the single Mendelian locus *S22* based on genetic mapping (Sobrizal et al., 2000). Although *S22A* and *S22B* harbor the same domain, DUF1668, the DUF1668-containing proteins at *S22A* and *S22B* are not functionally redundant. Phylogenetic analysis of DUF1668-containing sequences in Poaceae revealed that gene duplication and diversification of DUF1668-containing genes occurred in ancestral species of Poaceae, and the sterility-causing genes *S22A* and *S22B* belong to a specific clade, Clade 1 (Figure 3A). Since Clade 1 contains both species from the BOP and PACMAD clades (including the genera *Setaria*, *Panicum*, *Botryococcus*, and *Oryza*), ancestral DUF1668-containing genes likely originated before divergence of the BOP and PACMAD clades and are thus shared among these species. Since *S22A_j1*, *S22A_j2*, and *S22A_j3* also formed a monophyletic clade within Clade 1 (Figure 3B), they likely originated from gene duplications at *S22A* after divergence of the genus *Oryza*. Therefore, the genetic functions of these DUF1668-containing gene copies at *S22A* are likely to be redundant, and transformation of these copies restored the fertility of sterile pollen grains carrying *S22A-glum^s^*. By contrast, gene duplication between *S22A* (*S22A_j1*, *S22A_j2*, and *S22A_j3*) and *S22B* may have at least an ancestral population of the divergence of the BOP and PACMAD clades. Although this study demonstrated that genetic variants at both *S22A* and *S22B* induce HI, it will be necessary to further examine whether the DUF1668 domain has evolved as an HI factor in other Poaceae species.

### Molecular Behavior of S22B

*S22B* transcript levels were reduced in the order of homozygotes for *S22B-T65^+^*, heterozygotes, and homozygotes for *S22B-glum^s^* in the anthers at each of the unicellular, bicellular, and mature stages using forward and reverse primers targeting the same sequences at *S22B_j* and *S22B_g* (Figure 1E). These data suggest that the expression level of *S22B_g* is lower than that of *S22B_j*. The expression level of *S22B* also decreased as male gametogenesis progressed. The *S22B* transcripts are likely mainly contributed from male gametophytes rather than from sporophytic tissues (anthers) based on the results of the promoter-*GUS* assay (Figure 1F). On the other hand, *S22B* broadly expressed in vegetative tissues in RiceXpro expression database (Supplementary Figure S6; Sato et al., 2013). Comparison of the genomic regions of *S22B_j* and *S22B_g* revealed many nucleotide substitutions in the promoter region and the 5' untranslated region near the transcriptional start site. These substitutions may be involved in regulating interspecific differences in transcription between the two alleles.

Western blotting showed that the level of S22B protein increased with the progression of male gametogenesis, in contrast to the decrease observed at the transcription level (Figure 1G). No obvious difference in S22B protein accumulation was observed between the two homozygotes for *S22B-T65^+^* and *S22B-glum^s^*. These data suggest that the level of S22B protein is under control by a post-translational regulation mechanism or that *S22B-glum^s^* is sufficient for male gametogenesis. By contrast, the level of S22B protein was reduced in heterozygotes (Figure 1G). As one example of reduced expression only in heterozygotes in HI systems, genetic variants of a single gene between two diverged alleles at the *Sc* locus were reported to constitute the HI system (Shen et al., 2017). The japonica allele *Sc-j* contains a pollen-essential gene, and the indica allele *Sc-i* contains two or three tandem duplicates of an *Sc-j* homolog with a distinct promoter. In *Sc-j/Sc-i* hybrids, the high expression level of *Sc-i* in sporophytic cells causes suppression of *Sc-j* expression in pollen and selective abortion of *Sc-j*-pollen (Shen et al., 2017). Their study further revealed that feedback-mediated regulation of genes or proteins may result in the misregulation of gene expression between differentiated alleles in heterozygotes. Similar to the *Sc* system, the reduced S22B protein level in the anthers of heterozygotes observed in the present study could be due to allelic suppression of S22B protein *via* incompatible feedback modulation between the two alleles.

Our western blotting analysis of S22B also revealed that the 46 kDa band appeared specifically at the mature stage. In S22B_SS, development of pollen grains carrying the *S22B-glum^s^* allele starts to delay from the late bicellular stages, and this delay is particularly apparent at the mature stage as compared with normal genotypes (Sakata et al., 2014). Almost all of the pollen grains carrying the *S22B-glum^s^* allele could reach the tricellular stage but did not complete the formation of the male germ unit and failed to produce the pollen tube. We speculate that the reduction of the 46 kDa band in heterozygotes is involved in pollen semi-sterility. It is possible that the 46 kDa band resulted from alternative splicing of *S22B_j* and/or *S22B_g*, or from post-translational modification of the 37 kDa band, such as phosphorylation, lipidation, or glycosylation. The genetic effects of nucleotide substitutions and the 2-bp deletion on the *S22B* coding sequence for protein modification, and the mitochondrial localization of S22B have not yet been elucidated. Thus, these biochemical properties require further study.

In the linkage analysis of *S22A* (*n* = 3,072), homozygotes for *S22A-glum^s^* were not obtained (Supplementary Figure S2). The total seed set of self-pollinated *S22A*_SS plants was fertile, and self-pollinated seeds of *S22A*_SS showed normal seed germination. These results demonstrate that the *S22A-glum^s^* allele is insufficient for male gametogenesis in heterozygotes. Alternatively, the *S22B-glum^s^* allele is transmitted *via* male gametophytes in heterozygotes and homozygotes, which segregates at low frequency. The *S22A-glum^s^* allele encodes a truncated protein with loss of the complete DUF1668 domain (Figure 2C). In contrast, the *S22B-glum^s^* allele has a few single nucleotide substitutions and a 2-bp frameshift mutation at the C-terminal, but the DUF1668 domain was predicted to exist in the Pfam search (Finn et al., 2014). *S22B_m* is likely a loss-of-function allele, and a homozygous plant for the *S22-mer* allele segregated and showed normal pollen fertility (Supplementary Figure S4). Therefore, an unidentified causative mutation in *O. glumaepatula* may inactivate gene function, although the DUF1668 domain was predicted *in silico*.

### Hybrid Incompatibility at *S22A* and *S22B*

Diverged haplotypes, including multiple tightly linked genes, are known to induce HI in intra-specific and interspecific hybrids of rice, such as *Sa*, *S5*, and *S1*. The incompatible gene complex includes sporophytic genetic factors and gametophytic genetic factors that determine pollen fertility of its own gametophytes in heterozygotes. The BLAST similarity search using the cloned genes at the known HI as a query did not find homologous sequences within the *S22A* and *S22B* mapping regions. If HI systems conferred by *S22A* and *S22B* also evolve incompatible haplotypes to induce HI in hybrids, the genes cloned in this study only represent a portion of gametophytic members acting as a protector and were not sufficient to induce HI. To further identify other possible genes including a killer factor involved in HI at *S22A* or *S22B*, it is necessary to conduct defective mutant experiments using genome-editing methods such as CRISPR/Cas9 in heterozygotes showing HI. As suggested in the BDM model, nucleotide variants causing HI are heterozygous only at birth, which is necessary to escape from negative selection due to their own maladaptive phenotypes owing to this incompatibility. When *S22A_j1*, *S22A_j2*, and *S22A_j3* are considered as the ancestral types and the truncated genes at *S22A-glum^s^* are considered as the variant types, nucleotide variants of *S22A-glum^s^* occurring only at birth need to escape from natural selection *via* BDM partners such as duplicated genes or interacting genes in other genomic regions of *O. glumaepatula* or ancestral species. Since near-isogenic lines of *S22A* and *S22B* in the T65 genetic background were used in this study, the genetic phenomena may appear as monogenic events, and other BDM partners have not yet been identified.

In contrast, a single gene can also cause an HI system. Allelic suppression of the japonica allele at the *Sc* locus results from the feedback-mediated regulation of gene expression between differentiated alleles in inter-subspecific hybrids between japonica and indica rice (Shen et al., 2017). If the allelic suppression occurred on the semi-sterile *S22B*_SS, transcription level on heterozygote would show half of the homozygotes for *S22B-T65^+^* because transcription of *S22B-glum^s^* is suppressed on heterozygote. However, expression level on the *S22B*_SS was closed to average of homozygotes of *S22B-T65^+^* and *S22B-glum^s^*, suggesting that allelic suppression has not occurred on transcription level. Instead, we suggest that alternative splicing of S22B_j and/or S22B_g or production of the 46 kDa band resulted from post-translational modification is suppressed in pollen grains harboring *S22B-glum^s^* in allele-specific manner. The further studies may reveal this opinion. Another possibility may be that copy number variation of functional copies of DUF1668-containing genes in pollen grains results in competitive transmission efficiencies between fertile and sterile alleles. The allelic differences in development and starch absorption capacity may also result in the biased allocation of nutrient resources between gametophytes, leading to the distorted transmission efficiency of alleles.

In summary, HI at *S22A* and *S22B* could be caused by gene complexes within the candidate region, or by structural changes in a single gene. Further genetic analysis to identify other genetic loci interacting with *S22A* or *S22B*, and biochemical and genetic characterization of DUF1668-containing genes may reveal the HI mechanism *via* DUF1668.

## Data Availability Statement

The original contributions presented in the study are included in the article/Supplementary Material, further inquiries can be directed to the corresponding author.

## Author Contributions

MS: investigation, development of genetic materials, review, writing draft manuscript, and editing of the manuscript. NS and YM: investigation and development of plant materials. HK, JW, and TM: investigation and sequencing. HY and AY: project administration, funding acquisition, and supervision. YY: conceptualization, methodology, investigation, data curation, and writing draft manuscript. All authors contributed to the article and approved the submitted version.

### Conflict of Interest

The authors declare that the research was conducted in the absence of any commercial or financial relationships that could be construed as a potential conflict of interest.

## Footnotes

^1^http://hmmer.org/

^2^http://tree.bio.ed.ac.uk/software/figtree/
