# Supplementary material for: Domain Unknown Function DUF1668-Containing Genes in Multiple Lineages Are Responsible for F1 Pollen Sterility in Rice
Source: Front Plant Sci. 2021 Jan 26;11:632420. doi: 10.3389/fpls.2020.632420 (PMC7870705; doi:10.3389/fpls.2020.632420)
Supplement: Supplementary file 1 [file Data_Sheet_1.pdf]

Supplementary Materials

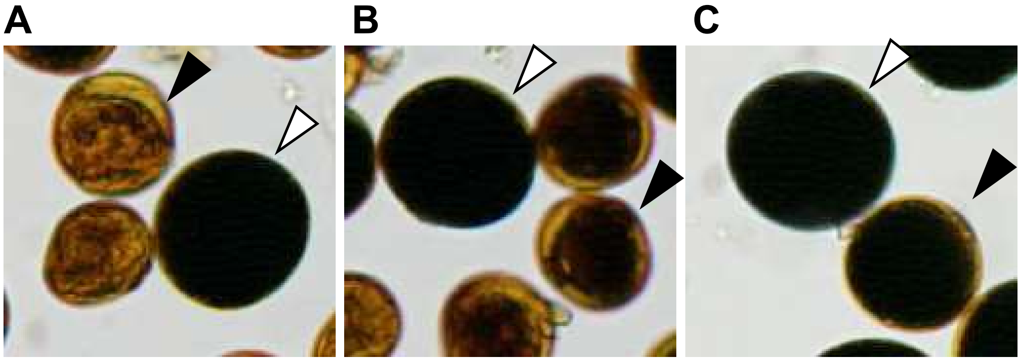

Supplementary Figure 1. Pollen morphology stained by I_2-KI at mature stage. (**A**) Pollen from the *S22A+S22B*_SS plant. (**B**) Pollen from the *S22A*_SS plant. (C) Pollen from the *S22B*_SS plant. Normal and abnormal pollen grains were represented by white and black arrowheads.

S22B

Marker / Recombinant	*RM12317*	*S22A*	*RM12329*	*M37*	*M48*	*M54*	*M39*	*M40*	*M42*	*M46*	*M26*	*RM279*	Pollen fertility	Abnormal pollen phenotype
9−7	H	H	H	H	H	H	H	H	H	T	T	T	Semi-sterile	*S22A*+*S22B* type
23−2	T	T	T	T	T	T	T	T	T	T	H	H	Normal	−
28−1	T	T	T	T	T	T	T	T	T	H	H	H	Normal	−
38−3	H	H	H	H	H	T	T	T	T	T	T	T	Semi-sterile	*S22A* type
39−7	T	T	T	T	H	H	H	H	H	H	H	H	Semi-sterile	*S22B* type

Supplementary Figure S2. The five informative recombinants for high-resolution mapping of *S22B*. Genotypes of *S22A* and *S22B* were discriminated from morphology of abnormal pollen phenotypes as *S22A*+*S22B* type, *S22A* type, and *S22B* type as shown in Supplementary Figure S1. When abnormal pollen on semi-sterile plants were *S22A*+*S22B* type, its pollen semi-sterile plants were estimated heterozygous both at *S22A* and *S22B*. Similarly, when abnormal pollen on semi-sterile plants were *S22A* type or *S22B* type, its pollen semi-sterile plants were estimated heterozygous at *S22A* or *S22B*, respectively. T, H, and G represent genotypes homozygous for the T65 allele, heterozygous, and homozygous for the IRGC105668 allele, respectively.

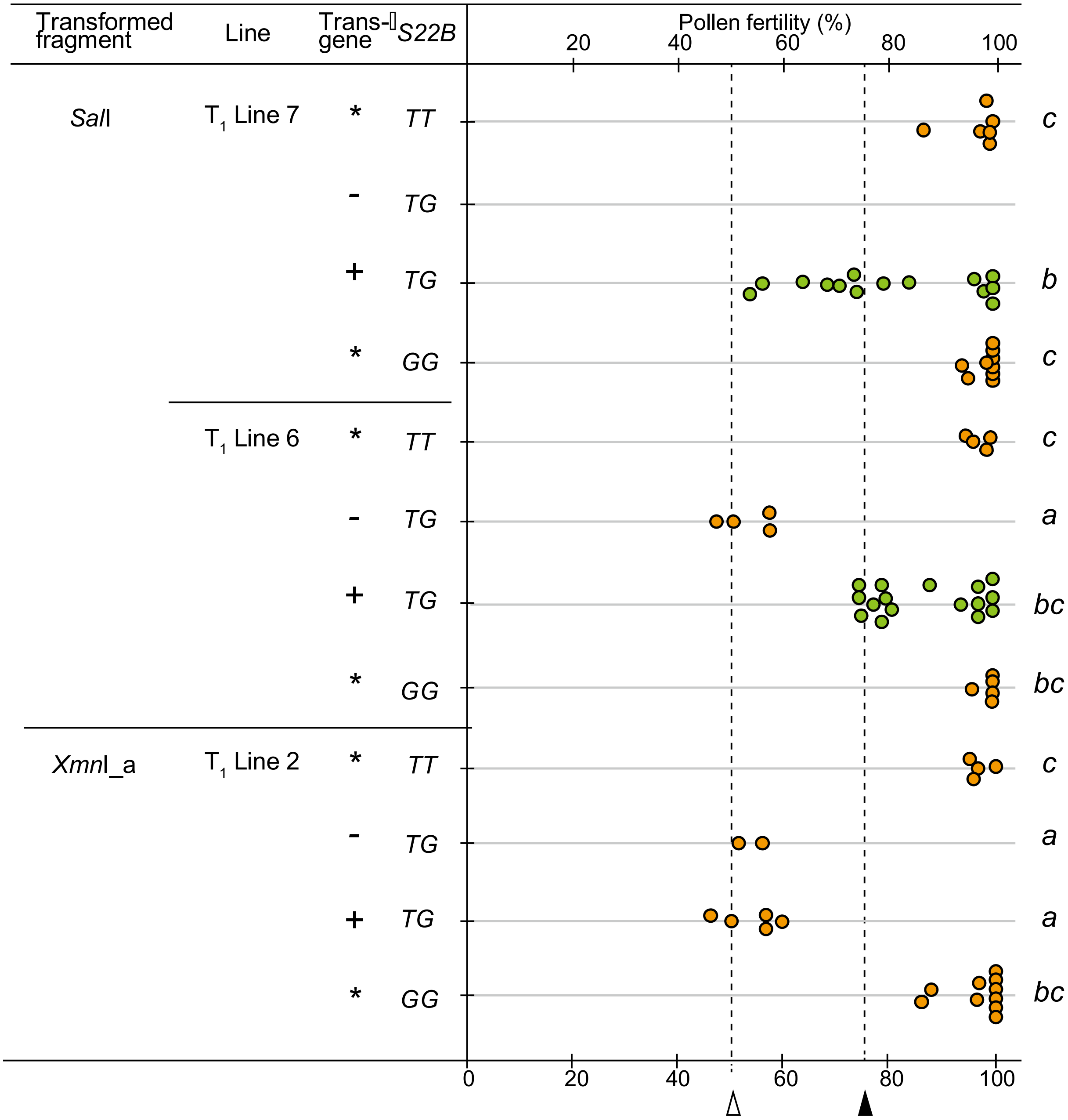

Supplementary Figure S3. Pollen fertility in the T_1 lines transformed by *Sal*I or *Xmn*I_a fragment. The two T_1 lines, 6 and 7, were derived from the two independent T_0 plants carrying a single copy and more than two copies of the *Sal*I fragment (transgene), respectively. + or - represent the T_1 plants with or without the transgene. Asterisk (*) represents unknown genotypes for the transgene. Black and white arrowheads indicate 75% and 50% pollen fertility, respectively. The heterozygotes harboring *Sal*I fragment showed increased pollen fertility as compared with null segregants of heterozygotes at *S22B* or the heterozygotes harboring *Xmn*I_a fragment. A pair of groups sharing the same alphabet indicate not significant difference in pollen fertility at 5% level of type I error (alpha) in Tukey-Kramer HSD test.

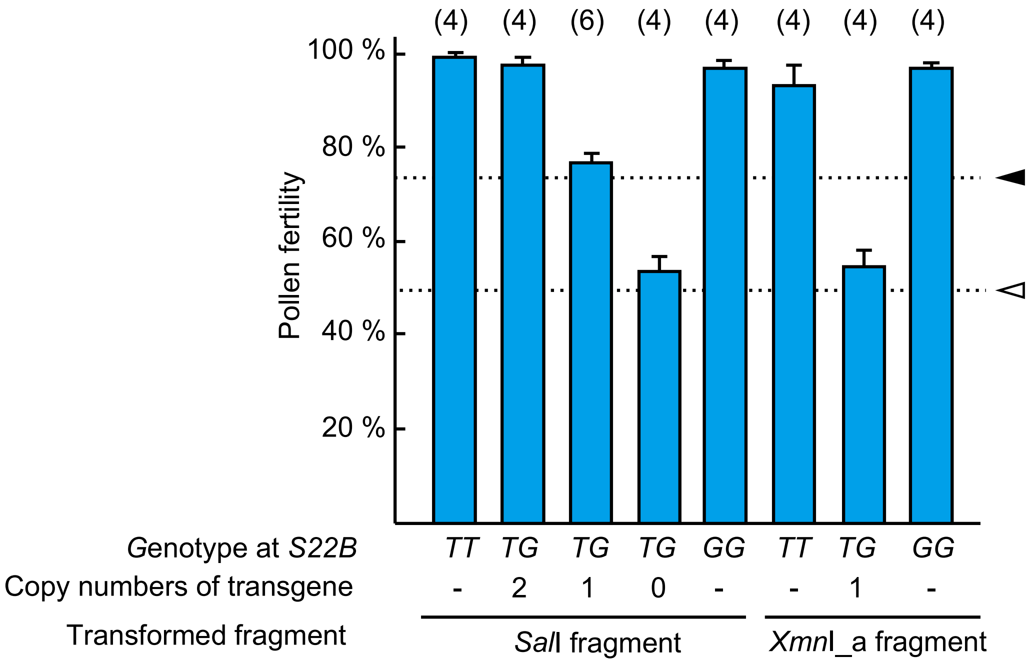

Supplementary Figure S4. Pollen fertility of the T_1 plants in the Line 6 and 2 derived from the T_0 plant with a single copy of the *Sal*I fragment or *Xmn*I_a fragment. The observed numbers of plants were indicated in parenthesis. *TT*, *TG*, and *GG* represent the homozygous for T65 (*Oryza sativa*), heterozygous and homozygous for IRGC105668 (*O. glumaepatula*), respectively. The T_1 plants heterozygous at *S22B* with two or one copies of the *Sal*I fragments correspond to homozygous or hemizygous for the transgene and recovered pollen fertility at approximately 100% or 75% (a black arrowhead) whereas the null T_1 segregants of the *Sal*I fragment showed pollen semi-sterility (a white arrowhead). The recovery of pollen fertility had not been observed by transformation of *Xmn*I_a fragment.

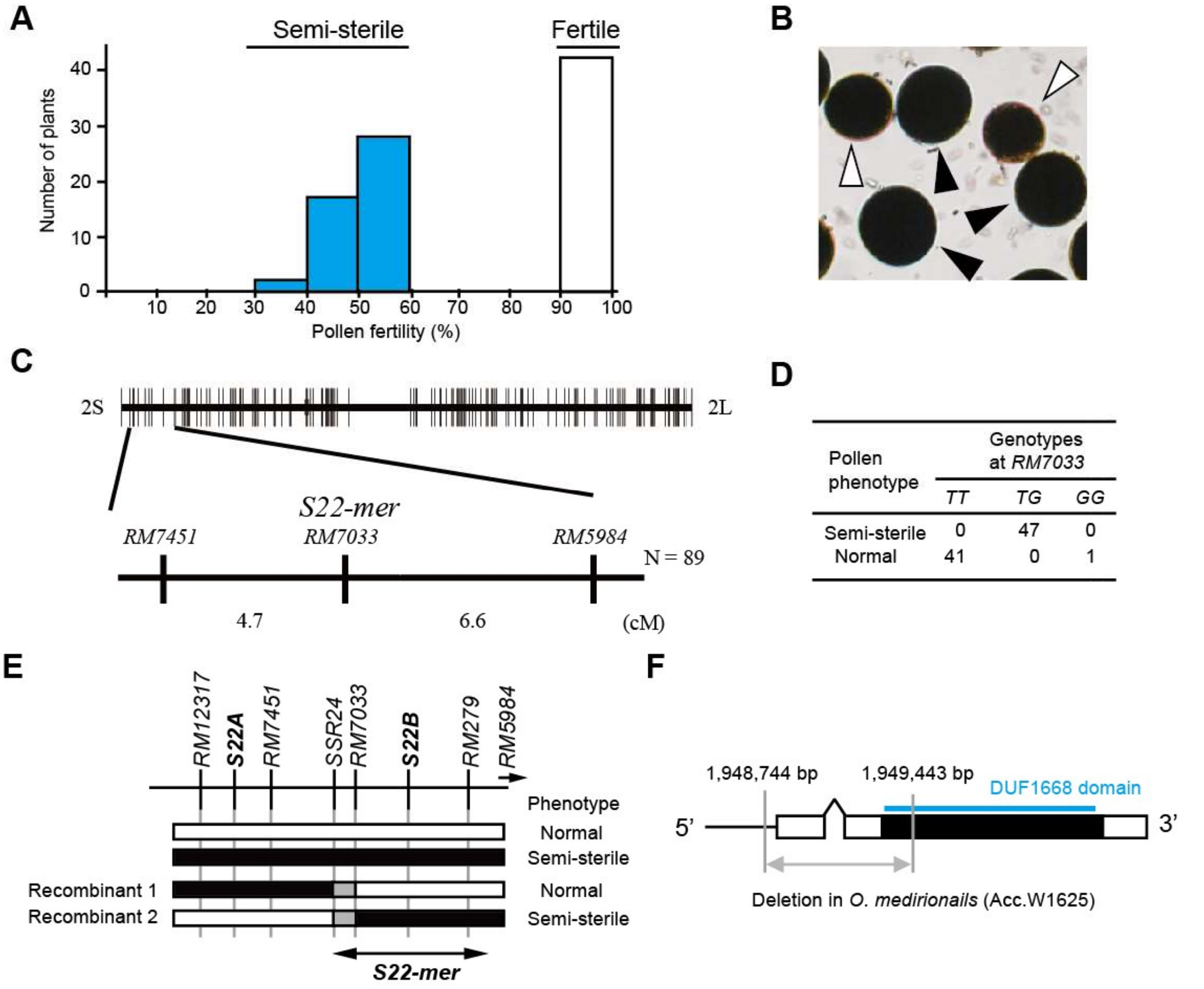

Supplementary Figure S5. Identification of *S22-mer*. (**A**) Frequency distribution of pollen fertility in a BC_4F_3 population derived from a cross between *Oryza sativa* L. cultivar Taichung 65 (T65) and *O. meridionalis* Ng accession W1625. (**B**) Morphology of sterile pollen on heterozygous plants at *RM7033*. Sterile pollen grains were stained by I_2-KI and similar to sterile pollen grains in *S22B*_SS plants. (**C**) Linkage map of *S22-mer*. (**D**) Linkage analysis of pollen semi-sterility with genotypes at DNA merker *RM7033*. (**E**) Genetic dissection of *S22-mer* genomic region. *S22-mer* responsible region were delimited between *SSR24* and *RM5984* containing *S22B* locus. (**F**) Genomic sequence at *S22B* in W1625. W1625 (*O. meridionalis*) lacks half of upstream region of *S22B*, suggesting that *O. meridionalis* allele at *S22B* is a loss-of-function allele.

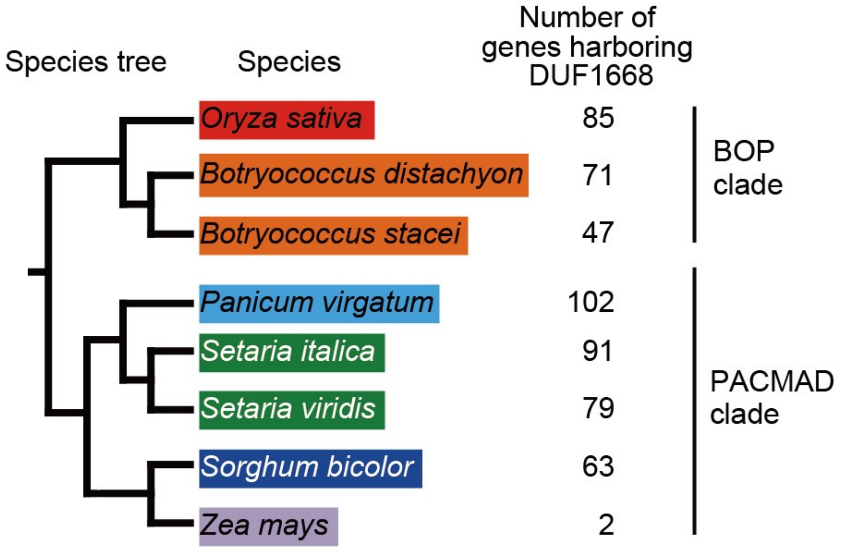

Supplementary Figure S6. Number of genes harboring DUF1668 domain in Poaceae species. In Phytozome 12 database (Goodstein et al. 2012), Genome and proteome database for *Setaria viridis*, *Setaria italica*, *Panicum virgatum*, *Botryococcus distachyon*, *Sorghum bicolor*, *Zea mays*, *Botryococcus stacei*, and *Oryza sativa* were available in Poaceae species.

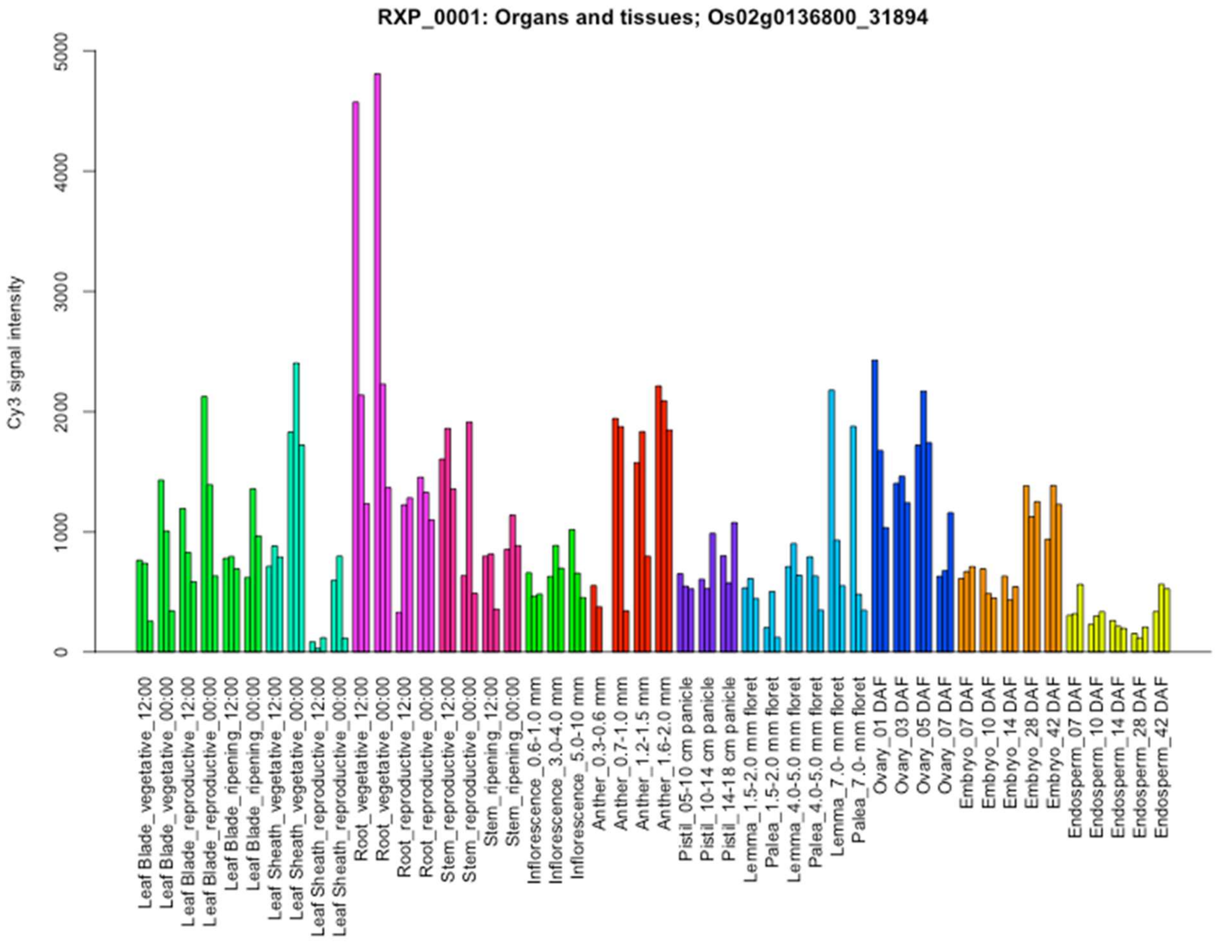

Supplementary Figure S6. Expression of *S22B* in other plant organs in RiceXpro database,.

1.1 Supplementary Table

Supplementary Table S1. PCR primers used in this study.

Marker	Forward primer sequence	Reverse primer sequence	Chr.	Nipponbare genome[1]			Marker types[2]	Reference
				Start	End	Size		
RM12317	ATAATTGTGGTCCCGAGTCATCC	TGCTTCTTCAACTCAACTCTCAGC	2	198,933	199,205	273	SSR	IRGSP(2015)
SSR5	TCCAGGTCAACACCTTACCC	CAGTGACACCACCACTCAGC	2	296,218	296,381	164	SSR	In this study
SSR14	TCTCCTTGAACAGCCAAACA	TGTTCTTGGCAAGCTCTCCT	2	386,085	386,267	183	SSR	In this study
M21	AAGAGAAGGTTGGGCTACGC	AAATGCATGCTTCCCTCATC	2	391,062	391,244	183	Indel	In this study
M24	TTTTAGTGCCAAGTGAAGGTG	TTCCGAACTCTTAGCTGATATGC	2	400,182	400,344	163	SNP	In this study
M31	TCTGTAATTCTGCATCATGTTCAA	TCCATTCGCACACAAGTAATG	2	420,067	420,169	103	Indel	In this study
SSR9A	ATCGTGATCGTGATCGATGG	GCTAGGGGGTGTGCTACAAG	2	426,590	426,747	158	SSR	In this study
SSR23	GCATGCACTGCAGATACCAA	TTTGCAGCAGAGCAGAAAAA	2	426,801	426,999	199	SSR	In this study
SSR9B	ATCGTGATCGTGATCGATGG	GCTAGGGGGTGTGCTACAAG	2	493,786	493,943	158	SSR	In this study
SSR33	CACCACCACCACCATCATC	GCGTGTGCTACTGACAACGTA	2	551,473	551,564	92	SSR	In this study
SSR35	ATATCTGGGCCTCCCAAAAG	TTCTTCTCCGCTTCTGCTTC	2	559,624	559,730	107	SSR	In this study
SSR37	CAACCGCCGAAAAGTAGTTC	GCTGTTGCAAGGCCTTATCT	2	565,707	565,797	91	SSR	In this study
RM12329	GACGGCATCATTCTGTCAGG	TCAGATGACTCCTTATCTGC	2	571,565	571,710	146	SSR	IRGSP(2005)
RM7451	TAATACGAGCAGCGATCGTG	GCTAATTGCAGCTTGTGTCG	2	651,843	651,996	154	SSR	IRGSP(2005)
RM12350	CGCAGCATATCAACCAATCACC	AGGAAAGAGAGCGGGAAGAATCC	2	1,040,535	1,040,678	144	SSR	IRGSP(2005)
SSR24	ATTCTCTTGGGAGTCCTAAC	GTCTGGGTACCGATGATCAC	2	1,078,157	1,078,473	317	SSR	In this study
RM7033	GTGCCCAACACTGCACTAAC	GTTGGCGGTGATTTCTGATG	2	1,660,838	1,660,979	142	SSR	McCouch et al. (2002)
M37	CCTAGTGTACACCGCTCTGG	TATGTGCATTCGTTGGCTCG	2	1,928,481	1,929,447	967	SNP	In this study
M48	CTCAACCAGCACAATCAATG	TCGCTGCTGCGATTCCACAG	2	1,931,482	1,932,618	1,137	Indel	In this study
M54	TGGAGATCAACCAGAACCAG	GTTGCTATGAGTATTGGACG	2	1,937,419	1,938,240	822	SSR	In this study
M39	CACCTGCATAACGATGAGGC	CCACAAGTTAGGTTTGCAAG	2	1,938,162	1,939,216	1,055	SSR	In this study
M40	TATTCGACATGCATGATGAG	TCAACCTGGGACTGTGGTAG	2	1,944,021	1,945,250	1,230	CAPS (DdeI)	In this study
M42	TTGAGCCTCATGTTCCATCG	GGTGAGTTGATCATGAGCTG	2	1,946,228	1,947,195	968	SSR	In this study
M46	GTTATCCAGACGAGGAGTGG	GTTTGCCACTGAGACTATTCG	2	1,949,650	1,950,749	1,100	SSR	In this study
M26	CTGTGGACAATTGGTGAAGC	GATGACTGCACAAGCTAGCG	2	1,950,664	1,951,743	1,080	CAPS (MvaI)	In this study
RM279	GCGGGAGAGGGATCTCCT	GGCTAGGAGTTAACCTCGCG	2	2,882,054	2,882,217	164	SSR	Temnykh et al. (2000)
RM5934	CTGTCGTGTCATGGAGCAAG	TGGAGAGGCAAGAAGGTTTG	2	3,487,361	3,487,459	99	SSR	McCouch et al. (2002)

[1]Positions in the reference sequence of Nipponbare (Os-Nipponbare-Reference-IRGSP-1.0 pseudomolecules).
[2]SSR and CAPS represent simple sequence repeats and cleaved amplified polymorphic sequences, respectively. The restriction enzyme for the CAPS markers are shown in parentheses.
[3]References Temnykh, S., W. Park., N. Ayres, S. Cartinhour, N. Hauck, L. Lipovich, Y. Cho, T. Ishii, S. R. McCouch (2000) Mapping and genome organization of microsatellite sequences in rice (*Oryza sativa* L.). Theor. Appl. Genet. 100: 697–712.
McCouch, S. R., L. Teytelman, Y. Xu, K. Lobos, K. Clare, M. Walton, B. Fu, R. Maghirang, Z. Li, Y. Xing, Q. Zhang, I. Kono, M. Yano, R. Fjellstrom, g. DeClerck, D. Schneider, S. Cartinhour, D. Ware, L. Stein (2002) Development and mapping of 2240 new SSR markers for rice (*Oryza sativa* L.). DNA Res. 9: 199–207.
IRGSP (2005) The map-based sequence of the rice genome. Nature 436:793–800.

Supplementary Table S2. Frequency of genotypes at *SSR23* by the T_1 generation for the complementation test.

Transgene	T_1 line	Each line						Subtotal			Total	k [2]
		TT [1]		*TG*		*GG*		*TT*	*TG*	*GG*		
		-	+	-	+	-	+					
S22A_j1	105	2	5	1	5	0	0	54	57	17	128	0.239
	103	2	5	1	2	0	1					
	3	5	8	4	12	0	3					
	104	0	2	2	6	0	3					
	5	3	12	3	8	0	4					
	4	2	8	2	11	0	6					
S22A_j2	6	8	5	4	13	0	0	19	20	0	39	0
	7	1	5	1	2	0	0					
S22A_j3	8	3	11	4	9	0	0	46	55	2	103	0.042
	108	0	1	0	6	0	0					
	109	0	4	0	7	0	0					
	10	1	7	1	6	0	1					
	9	6	13	6	16	0	1					
Empty	1	5	8	2	9	0	0	35	41	0	76	0
	2	1	5	3	11	0	0					
	11	2	3	0	6	0	0					
	101	0	8	1	5	0	0					
	102	1	2	2	2	0	0					

[1] *TT*, *TG*, and *GG* are homozygous for *S22A-T65*$^{+}$, heterozygous, and homozygous for *S22A-glum*^{s}, respectively.

[2] *k* represents the transmission efficiency of *S22A-glum*^{s} via pollen from maximum-likelihood estimation.
