# Supplementary material for: Domain Unknown Function DUF1668-Containing Genes in Multiple Lineages Are Responsible for F1 Pollen Sterility in Rice
Source: Front Plant Sci. 2021 Jan 26;11:632420. doi: 10.3389/fpls.2020.632420 (PMC7870705; doi:10.3389/fpls.2020.632420)
Supplement: Supplementary file 2 [file Data_Sheet_2.pdf]

Supplementary Materials

1 Supplementary methods

Maximum likelihood estimation of transmission frequency of alleles

Here we assume allele *A* and *a* at a single locus with male transmission frequency of *1-k* and *k* (0<*k*<1) and female transmission frequency of 1/2 and 1/2, respectively. In self-pollinated progenies of a heterozygous plant, genotypic frequency of *AA*, *Aa*, and *aa* are expected in a ratio of (1-*k*)/2 : 1/2 : *k*. When we observed O_{AA}, O_{Aa}, and O_{aa} numbers of *AA*, *Aa*, and *aa* genotypic plants in the self-pollinated progenies of the heterozygous plant, likelihood function of *k*, *L*(*k*), when observation are given is as a function of multinomial probability

$$L(r) = \frac{n!}{O_{AA}! \cdot O_{Aa}! \cdot O_{aa}!}\left(\frac{1-k}{2}\right)^{O_{AA}} \cdot \left(\frac{1}{2}\right)^{O_{Aa}} \left(\frac{k}{2}\right)^{O_{aa}}$$

where $O_{AA} + O_{Aa} + O_{aa} = n$. The maximum likelihood estimate of *k* ($k = \hat{k}$) is given by

$$\frac{\mathrm{d}}{\mathrm{d}k} l(k) = 0$$

where $l(k)$ is log likelihood function of *L*(*k*) ($l(k) = \log\left(L(k)\right)$). By solving this equation, transmission efficiency is provided as an simple equation

$$\hat{k} = \frac{O_{aa}}{O_{AA} + O_{aa}}$$

as the maximum likelihood estimate $\hat{k}$.

In the body text, we defined the T65 and IRGC105668 alleles as *A* and *a* alleles, respectively.

2 Supplementary Data

```
S22B_m          ------------------------------------------------------------
S22B_J          TCAAAATCATCTTGTCTGAAATCAAAATCTTGGTGACAACTGACAAGTGTTGCAAAAAAA
S22B_g          TCAAAATCATCTTGTCTGAAATCAAAATCTTGGTGACAACTGACAAGTGTTGCAAAAAAA


S22B_m          ------------------------------------------------------------
S22B_J          TACCGTCTTGTGGGCTCGTGAAGGGGCCAAAGAACGGGATGGGGAATGGAAACGCGCGAA
S22B_g          TACCGTCTTGTGGGCTCGTGAAGGGGCCAAAGAACGGGATGGGGAATGGAAACGCGCGAA


S22B_m          ---------------AGGAGAGGAGCAGCACCAAGATCTCCAGCCTGAACTGCAT--CGC
S22B_J          GCAGCTGAAGCGAGCAGGAGAGGAGCAGAACCAAGATCACCAGCCTGAACTGCATTGTGC
S22B_g          GCAGCTGAAGCGAGCAGGAGAGGAGCAGAACCAAGATCACCAGCCTGAACTGCATTGTGC
                               ************* ********* ****************   **

S22B_m          TTCCAATTGGTTTGCTGATCGAATCAAGAAATCTTCGAGGTAAAAATGGTGGTGAGAACT
S22B_J          TTCCAATTGGTTTGCTGATCGAATCTAGAAATCTTCGAGGTAAAAATGGTGGTGAGAACT
S22B_g          TTCCAATTGGTTTGCTGATCGAATCAAGAAATCTTCGAGGTAAAAATGGTGGTGAGAACT
                ************************* **********************************

S22B_m          GAGAACAGCATCGATCAGTGTAAGCCAGGTGCATTCCTGATCCTTCTTCTCTCTGTGGCA
S22B_J          GAGAACAGCATCGATCAGTGTAAGCCAGGTGCACTCCTGATCCTTCTTCTCTCTGTGCCA
S22B_g          GAGAACAGCATCGATCAGTGTAAGCCAGGTGCACTCCTGATCCTTCTTCTCTCTGTGCCA
                ********************************* *********************** **
```

```
S22B_m          ATTTACATGGAGCAAGCAGTAGTGAACTAGTACTGATCACTGAAGCTTGCATTATCATGC
S22B_J          ATTTATATGGAGCAAGC-------------------------AAGCTTGCATTATAATGC
S22B_g          ATTTATATGGAGCAAGCAGTAGTGAACTAGTACTGATCACTGAAGCTTGCATTATAATGC
                ***** ***********                         ************* ****

S22B_m          CAATTAATTAAAGCTGATAAGATGTTGATTTGCATGGATTAAGCTATGATTGTAGTTAAC
S22B_J          CAATTAATTAAAGCTGATAAGATGTTGATTTGCATGGATTAAGCTATGATTGTAGTTAAC
S22B_g          CAATTAATTAAAGCTGATAAGATGTTGATTTGCATGGATTAAGCTATGATTGTAGTTAAC
                ************************************************************

S22B_m          GTTAATTTCTGGTCACCCACCTGTTGACCACCATCACCCCTCCTTGTCTGGATAACTTAT
S22B_J          GTTAATTTCTGGTCACCCACCTGTTGACTACCATCACCACTCCTCGTCTGGATAACTTAT
S22B_g          GTTAATTTCTGGTCACCCACCTGTTGACTACCATCACCACTCCTCGTCTGGATAACTTAT
                **************************** ********* ***** ***************

S22B_m          CTGAGTTGCGTGGAGCATCCATTGCTTTTGTTGGTACGTTTCT-------------CAAG
S22B_J          CTGAGTTGCGTGGAGCATCCATTGCTTTTGTTGGTACGTTTCT-------------CAAG
S22B_g          CTGAGTTGCGTGGAGCATCCATTGCTTTTGTTGGTACGTTTCCTGCAGCAATGTCGCAAG
                ******************************************              ****

S22B_m          TTAACCCTCCTAGACGAAATCAACATGCAGGACTGTCAATTTGTCATTGCAAAAGGAGGG
S22B_J          TTAACCCTCCTAGACGAAATCAACATGCAGGACTGTCAATTTGTCATTGCAAAAGGAGGG
S22B_g          TTAACCCTCCTAGACGAAATCAGCATGCAGGATTGTCAATTTGTCATTGCAAAAGGAGGG
                ********************** ********* ***************************

S22B_m          GGAAAAAATAATCGCAGGATTGGTTTGAAAAAGGAAAATCAGGATATGGGAGACAAACTT
S22B_J          GGAAAAAATAATCGCAGGATTGGTTTGAAAAAGGAAAATCAGGATATGGGAGACAAACTT
S22B_g          GAAAAAAAGAATCGCAGGATTGGTTTGAAAAAGAAAAATCAGGAGATTGGAGACAGACTC
                * ****** ************************ ********** ** ******* ***

S22B_m          GAACCTTCGCTTTATGGGCCAAGT------------------------------------
S22B_J          GAACCTTCGCTTTATGGGCCTAGTCGGCCCTCGGCCCATTCGAGCCCAAACATAACCCTA
S22B_g          ACACCTTCGCTTTATGGGCCTAGTCGGCCCTCGGCCCATTCGAGCCCAAACATAATCCTA
                  ****************** ***


S22B_m          ------------------------------------------------------------
S22B_J          ACATAAGTCT--TGGTCCTTGTCCCTCGCAGCAGAATAAAAAATCGCCGCCGCCGC----
S22B_g          ACATAAGTCTTCTAGTCCTTGTCCCTCGCGGCAGTATAAAAAATCGCCGCCGCGGCAGTA

S22B_m          ------------------------------------------------------------
S22B_J          ---------------------CGCCGCC-GCCGCCGCTGCCCGTC------------CCC
S22B_g          TAAAAAATCGCTGCCGCCGTTCGCCGCCCGCCGCCGCTGACCGTCGCCTTCTCCGTCCCC

                   25 bp insertion                           12 bp insertion
S22B_m          ------------------------------------------------------------
S22B_J          GCAACCCAACGCCTGATTCGCTTCTCCATCTGCTTGCATTCAACCGGGTTGTGGAGGAGG
S22B_g          GCAACCCAACGCCTGATTCGCTTCTCCATCTGCTTGCATTCAACCGGGTTGTGGAGGAGG

S22B_m          ------------------------------------------------------------
S22B_J          AGTAGGAGAAATCCGGTGGATATCGATCGGATCCAGATGAACACTCGACTGGATTCCGGC
S22B_g          AGTAGGAGAAATCCGGTGGATATCGATCGGATCCAGATGAACACTCGACTGGATTCCGGG

S22B_m          ------------------------------------------------------------
S22B_J          GGACAGAAGTAAATCCGGAGTCGGTTTTTTCGTAAATCGCAGGTACTCTACGCAATTTTT
S22B_g          GGACAGAAGTAAATCCGGAGTCGGTTTTTTCGTAAATCGCAGGTACTCTACGCAATTTTT

S22B_m          ------------------------------------------------------------
S22B_J          GATCCTAATTGTTATACTAGTAGTAATACAGTGGGAGATGTGAGTAATAATTGCTGGATT
S22B_g          GATCCTAATTGTTATACTAGTAGTAATACAGTGGGAGATGTGAGTAATAATTGCTGGATT

S22B_m          ------------------------------------------------------------
S22B_J          ATTTCTGGTTTTTGTACAAGGAGAAATCGAGGTACAGATATTCATGCTGCCTCGATGTGT
S22B_g          ATTTCTGGTTTTTGTACAAGGAGAAATCGAGGTACAGATATTCATGCTGCCTCGATGTGT

S22B_m          ------------------------------------------------------------
S22B_J          TGGAATCAACAAGATGAACAATTAACATCCGAAGCACAAAGAATTTCGGATAAATCCAAC
S22B_g          TGGAATCAACAAGATGAACAATTAACATCCGAAGCACAAAGAATTTCGGATAAATCCAAC

S22B_m          ------------------------------------------------------------
S22B_J          TAATCGATCGATCGTATATGCAACTAGTGCATATACATATCTAGTAGATTCCTGTTCATA
S22B_g          TAATCGATCGATCGTATATGCAACTAGTGCATATACATATCTAGTAGATTCCTGTTCATA


S22B_m          ------------------------------------------------------------
```

```
S22B_J          ATTTGGAGGAGGAAGATCAGTTATGAGTTACCGGCGATTTGTGAATCTGGTGCTTGATAA
S22B_g          ATTTGGAGGAGGAAGATCAGTTATGAGTTACCGGCGATTTGTGAATCTGGTGCTTGATAA


S22B_m          ------------------------------------------------------------
S22B_J          GGTCCAAGGCAACTACACAGTGCGGCGCATGGACATGTCACGCTTCTTCCTCCCCCGTAA
S22B_g          GGTCCAAGGCAACTACACAGTGCGGCGCATGGACATGTCACGCTTCTTCCTCCCCCGTAA


S22B_m          ------------------------------------------------------CGGCAA
S22B_J          ATTGGCAACGCCGCTGGACGCCGGAGCACACGACGGCGCGGCGGCGGTGGAGTACGGCAA
S22B_g          ATTGGCAACGCCGCTGGACGCCGGAGCACACGACGGCGCGGCGGCGGTGGAGTACGGCAA
                                                                      ******

S22B_m          TCTGCCATGCCCGGTGATGAGCTTCCGTGCATCGGTCTGCGCGATGGAGACGATGGAGTT
S22B_J          TCTGCCATGCCCGGTGATGAGCTTCCGTGCATCGGTCTGCCCGATGGAGACGATGGAGTT
S22B_g          TCTGCCATGCCCGGTGATGAGCTTCCGTGCATCGGTCTGCGCGATGGAGACGATGGAGTT
                **************************************** *******************
                                                        ^ CCG(P)> GCG(A)

S22B_m          CATGCTCCTTGGTGGAAGGCATAACAAGATAGTCGGCACCGACCTGACGGGGCGCACTCT
S22B_J          CATGCTCCTTGGTGGAAGGCATAACAAGATAGTCGGCACCGACCTGACGGGGCGCACCCT
S22B_g          CATGCTCCTTGGTGGAAGGCATAACAAGATAGTCGGCACCGACCTGACGGGGCGCACCCT
                ********************************************************* **

S22B_m          CCTGTACGACCCCGACGAGCACGTCGTCCGCTCCCTGCCCACCCTCCCCATGCCCAAGTT
S22B_J          CCTGTACGACCCCGACGAGCACGTCGTCCGCTCCCTGCCCACCCTCCCCATGCCCAAGTT
S22B_g          CCTGTACGACCCCGACGAGCACGTCGTCCGCTCCCTGCCCACCCTCCCCATGCCCAAGGT
                ********************************************************** *
                                                           TTC(F)>GTC  (V)  ^

S22B_m          CTCCGCCGTGTCCCTCACCATCGGCGACGACGACCTTTACATCCTCGACGACATCCAGGG
S22B_J          CTCCCCCGTGTCCCTCACCATCGGCGACGACGACCTTTACATCCTCGACGACATCCAGGG
S22B_g          CTCCGCCGTGTCCCTCACCATCGGCGACGACGACCTTTACATCCTCGACGACATCCAGGG
                **** *******************************************************
                    ^ CCC(P)> GCC(A)

S22B_m          CCCGTTCACCGGCGGCCATGATCACTGCTTCCACGCCCTAACCTATAGTAGGGAGAGCTT
S22B_J          CCCGTTCACCGGCGGCCATGATCACTGCTTCCACGCCCTAACCTATAGTAGGGAGAGCTT
S22B_g          CCCGTTCACCGGCGGCCATGATCACTGCTTCCACGCCCTAACCTATAGTAGGGAGAGCTT
                ************************************************************

S22B_m          CACCGACGCCGACGGCGACTGGTGTTGCCACACTCTCCCGCCACCTCCTTACATGGTGAA
S22B_J          CACCGACGCCGACGGCGACTGGTGTTGCCACACTCTCCCGCCGCCTCCTTACATGGTGAA
S22B_g          CACCGACGCCGACGGCGACTGGTGTTGCCACACTCTCCCGCCGCCTCCTTACATGGTGAA
                ****************************************** *****************

S22B_m          GCAACGCGGCGACTTCCAATTCGACTCCTACGCGGTGGTGGATGGGGTGGACATCTGGAT
S22B_J          GCAACGCGGCGACTTCCAATTCGACTCCTACGCGGTGGTGGATGGGGTGGACATCTGGAT
S22B_g          GCAACGCGGCGACTTCCAATTCGACTCCTACGCGGTGGTGGATGGGGTGGACATCTGGAT
                ************************************************************

S22B_m          ATCCAAGCAAGGCGTCGGCACCTACAGGTTCCACACGGAGCGCGGAGAGTGGAGCGCGGT
S22B_J          ATCCAAGCAAGGCGTCGGCACCTACAGGTTCCACACGGAGCGCGGAGAGTGGAGCGCGGT
S22B_g          ATCCAAGCAAGGCGTCGGCACCTACAGGTTCCACACGGAGCGCGGAGAGTGGAGCGCGGT
                ************************************************************

S22B_m          GGCGAGCGGCGGCGAGTGGTGCGCGATGCCGTTCACCGGCCTCGCCGTGTACGTGCCGGA
S22B_J          GGCGAGCGGCGGCGAGTGGTGCGCGATGCCGTTCACCGGCCTCGCCGTGTACGTGCCGGA
S22B_g          GGCGAGCGGCGGCGAGTGGTGCGCGATGCCGTTCACCGGCCTCGCCGTGTACGTGCCGGA
                ************************************************************

S22B_m          GCACGGCCTGTTCTACGGCCTCGCGTCCGGCACCGACAACGTCCTATCTGCGTCTGATCT
S22B_J          GCACGGCCTGTTCTATGGCCTCGCGTCCGGCACCGACAACGTCCTATCTGCGTCTGATCT
S22B_g          GCACGGCCTGTTCTACGGCCTCGCGTCCGGCACCGACAACGTCCTATCTGCGTCTGATCT
                *************** ********************************************
                               ^ TAT(Y)> TAC(Y)

S22B_m          CATTAGCGGCGGGAGAAAGCCGGAGCAGCACAGCCTCCTTCCTCTGGAGTACACGCCGCC
S22B_J          CATTAGCGGCGGGAGAAAGCCGGAGCAGCACAGCCTCCTTCCTCTGGAGTACACGCCGCC
S22B_g          CATTAGCGGCGGGAGAAAGCCGGAGCAGCACAGCCTCCTTCCTCTGGAGTACACGCCGCC
                ************************************************************

S22B_m          CAAGGCGTTGACGCAGGTGTCTTCCCACCTCGTGCACCTGGGCTCTGCCAACTTCTGCAT
S22B_J          CAAGGCGTTGACGCAGGTGTCTTCCCACCTCGTGCACCTGGGCTCTGCCAACTTCTGCAT
S22B_g          CAAGGCGTTGACGCAGGTGTCTTCCCACCTCGTGCACCTGGGCTCTGCCAACTTCTGCAT
                ************************************************************

S22B_m          CGCCAGGTTCTTCGAGACCGGCTTCTTCGACGCCGACGAACAACACAACCCGGAGCTGTT
S22B_J          CGCCAGGTTCTTCGAGACCGGCTTCTTCGACGCCGACGAACAACACAACCCGGAGCTGTT
S22B_g          CGCCAGGTTCTTCGAGACCGGCTTCTTCGACGCCGACGAACAACACAACCCGGAGCTGTT
                ************************************************************

S22B_m          CGCTGTGTTCACCGCCGTCGAGGTGGAGCGCTGCGACGACACTGGAGTGCTCCGCCTCGT
```

```
S22B_J          CGCTGTGTTCACCGCCGTCGAGGTGGAGCGCTGCGACGACACTGGAGTGCTCCGCCTCGT
S22B_g          CGCTGTGTTCACCGCCGTCGAGGTGGAGCGCTGTGACGACACTGGAGTGCTCCGCCTCGT
                ********************************* **************************
                                                 ^ TGC(C)> TGT(C)

S22B_m          GAAGCACAAGTCGGAGATGTACAACCTTATCAGCGGAATATATTATTGGGTGCTTTAATC
S22B_J          GAAGCACAAGTCGGAGATGTACAAGCTTATCAGCGAAATATATTATTGGGTGCTTTAATC
S22B_g          GAAG--CAAGTCGGAGATGTACAAGCTTATCAGCGGAATATATTATTGGGTGCTTTAATC
                ****  ****************** ********** ************************
                    ^ 2bp deletion

S22B_m          AGCTATTACTCCCTCCGTTTCAGATTATATTTATTAAAATCAAATGCTTAATTTATATTG
S22B_J          AGCTATTACTCCCTCCGTTTCAGATTATATTTATTAAAATCAAATGCTTAATTTATATTG
S22B_g          AGTTATTACTCCCTCCGTTTCAGATTATATTTATTAAAATCAAATGCTTAATTTATATTG
                ** *********************************************************

S22B_m          GGTCCGTGAGGGGACAGTAAAGTTCTTTGCTTAATTTATATTTCTTATTTCCCAATTTCC
S22B_J          GGTCCGTGAGGGGACAGTAAAGTTCTTTGCTTAATTTATATTTCTTATTTCCCAATTTCC
S22B_g          GGTCCGTGAGGGGACAGTAAAGTTCTTTGCTTAATTTATATTTCTTATTTCCCAATTTCC
                ************************************************************

S22B_m          TTGTAAATCAGATGATATATTTTGCTTGGAGATATCATTGGTTTTTCCTTGTAAATCTTT
S22B_J          TTGTAAATCAGATGATATATTTTGCTTGGAGATATCATTGGTTTTTCCTTGTAAATCTTT
S22B_g          TTGTAAATCAGATGATATATTTTGCTTGGAGATATCATTGGTTTTTCCTTGTAAATCTTT
                ************************************************************

S22B_m          TTACTAATTCATATCATTGGTTTTTCCTGACT
S22B_J          TTACTAATTCATATCATTGGTTTTTCCTGACT
S22B_g          TTACTAATTCATATCATTGGTTTTTCCTGACT
                ********************************
```

Supplementary Data 1. Multiple alignment of genomic sequences at *S22B_j*, *S22B_g*, and *S22B_m*. Genomic sequences of *S22B_j* including promoters 700-bp upstream from transcription initiation sites and coding sequences (CDS) and 5'- and 3'- untranslated region (UTR). The homologous sequences from *S22B_g* and *S22B_m* were aligned with that of *S22B_j*. Red letters represent variants from ancestral sequences among three sequences. Sequences shaded by yellow, green, and orange represent 5'-UTR, CDS, and 3'-UTR, respectively. Locations of the nucleotide variations between *S22B_j* and *S22B_g* explained in Figure 2D were shown by hat letters (^).
